# Segmented profile analysis (SEPA): Plane-wise decomposition of within-person variation via ipsatized singular value decomposition

**DOI:** 10.3758/s13428-026-03102-0

**Published:** 2026-07-22

**Authors:** Se-Kang Kim, Joe Grochowalski

**Affiliations:** 1 Psychology Division, Department of Pediatrics, Baylor College of Medicine/Texas Children’s Hospital, Houston, TX 77030 USA; 2 College Board, New York, NY 10281 USA

**Keywords:** Biplot, Ipsatization, SVD/PCA, Person-centered profiling, Cosine similarity, Multidimensional profiles

## Abstract

Traditional profile analyses summarize multivariate person data with overall mean levels and relative patterns, but existing methods often blur these sources of variation or reduce each individual to a single best-fitting profile. Segmented Profile Analysis (SEPA) offers a unified, ipsatized singular-value decomposition (SVD) framework that decomposes individual profiles into orthogonal level (LE) and pattern (PE) effects and, crucially, introduces plane-wise segment profiles (summaries of each person's response pattern within each variable-contrast dimension) as primary person-oriented objects. Within each low-dimensional plane, SEPA defines a projected response pattern (segment profile), domain–person cosines that index variable-by-variable alignment, and plane-fit correlations that summarize how closely an individual’s pattern follows the plane’s domain structure. Across planes, SEPA aggregates information via singular-value weighting to yield an overall segment profile while preserving contrast-specific signal. A practical workflow combines ipsatized SVD, parallel analysis for PE dimensionality, marker-domain rules, bootstrap confidence intervals, and subspace-stability diagnostics. Using multivariate cognitive data from the Woodcock–Johnson IV, SEPA identifies interpretable marker domains, reveals distinct pattern facets across planes, and provides person-oriented indices that can be carried into standard regression models. Simulation studies examine the stability of segment profiles and cosines under varying sample sizes and variance structures. SEPA thus supplies a reproducible, geometry-based foundation for person-centered measurement that connects Q-type factor analysis, biplot methods, and contemporary within-person profiling in multidomain assessments.

**Supplementary Information:**

The online version contains supplementary material available at https://doi.org/10.3758/s13428-026-03102-0.

## Introduction

### Person-centered foundations

Quantitative psychology has long relied on R-type multivariate models that explain relationships among variables rather than among persons. Classical R-type factor analysis (Spearman, 1904) characterizes latent dimensions underlying covariation among observed measures, whereas Q-type factor analysis (Cattell, 1952, 1957) inverts this logic—decomposing covariation among individuals’ profiles across variables. The person-oriented perspective is especially valuable when the configuration of strengths and weaknesses within each individual—not merely their overall level—carries substantive meaning. In cognitive, clinical, and personality assessment, such within-profile structure often reveals heterogeneity that group averages conceal.

Profile-analytic methods, including profile analysis via multidimensional scaling (PAMS; Davison et al., 1996; Kim et al., 2004, 2007, 2017), factor-analytic profile analysis (FAPA; Davison et al., 2009; Kim, 2013, 2024; Weinshenker & Kim, 2022), and latent profile analysis (LPA; Asparouhov & Muthén, 2012; Ferguson & Hull, 2019; Gabriel et al., 2018; Spurk et al., 2020), were designed to model these within-person patterns. These approaches provide person-level representations—correlation-based correspondence weights (PAMS/FAPA) or class probability vectors (LPA)—that can be related to external variables. Yet they characterize within-person patterns primarily through a single dimension or class and do not directly represent the variable contrasts that give profiles their distinctive shape. When an individual's profile involves multiple simultaneous contrasts—for example, high processing speed alongside low verbal reasoning—a single dimension or latent class cannot capture both contrasts at once, and contrast-specific information is consequently masked, even in rich multivariate applications where profile methods have been used to study misophonia, symptom patterns, or linguistic profiles (e.g., McKay et al., 2018; Santi et al., 2024; Spencer et al., 2024).

### Motivation and scope

We introduce segmented profile analysis (SEPA), a variance-based, person-centered framework that reformulates profile analysis within the singular-value decomposition (SVD) family. SEPA preserves the familiar interpretability of principal component and biplot methods while providing plane-wise and cross-plane summaries that support transparent within-person inference. The method directly engages the level effect (LE) and pattern effect (PE) structure underlying profile analysis. Classical profile analysis distinguishes LE and PE in a primarily unidimensional manner—an approach supported conceptually and empirically in recent work (Kim, 2024; Kim & Kim, 2024)—but offers limited tools for decomposing, summarizing, and aggregating multiple pattern facets.

A useful way to understand SEPA is as a generalization of principal component analysis (PCA) that shifts focus from individual components to planes defined by pairs of components. Where classical PCA identifies a sequence of orthogonal components—each a single axis that captures the most remaining variance, orthogonal to all prior axes—SEPA identifies orthogonal planes, each defined by a pair of singular vectors. Just as successive components are ordered by the variance they explain individually, successive planes are ordered by their joint proportion of pattern variance. This plane-based perspective is not merely a cosmetic repackaging of PCA; it is the geometric structure needed to define within-person segment profiles, domain–person cosines, and plane-fit indices as primary, interpretable person-oriented indices.

The practical advantage of planes over individual components can be seen in a simple thought experiment. Suppose two individuals, Person A and Person B, both have large coordinates on Dimension 1 of the ipsatized SVD—they appear equally differentiated along that single axis and might seem to have similar profiles. When Dimension 2 is added and the two-dimensional plane is examined, however, Person A's coordinates fall in the upper-right quadrant (strongly aligned with CP and the positive pole of Dimension 2), while Person B's coordinates fall in the lower-left quadrant (aligned with VP and the negative pole). Their ipsatized profiles are, in fact, mirror images of each other—a CP-dominant pattern versus a VP-dominant pattern. This distinction is completely invisible in any single-dimension projection and can only be recovered by treating the two dimensions as a joint plane. SEPA makes this plane-wise representation the primary unit of analysis.

SEPA extends this framework by treating each two-dimensional latent plane obtained from ipsatized SVD as a coherent pattern facet and by defining new person-oriented quantities on that plane. By modeling LE and multiple PE facets within a geometric plane, SEPA yields graded, facet-specific alignment without class discretization—an advantage over latent profile analysis and related mixture approaches, especially when different latent facets dominate different planes in multidomain assessments.

### Conceptual geometry

Let $\mathbf{X}\in {\mathbb{R}}^{n\times p}$ denote a matrix of persons (rows) by domains (columns). SEPA begins by partitioning $\mathbf{X}$ into two orthogonal components:

$$\mathbf{X}=\mathbf{L}+\mathbf{P},$$

where $\mathbf{L}$ captures the level effect (LE)**—**each person’s overall elevation, computed as ${x}_{i\bullet }=\left(1/p\right)\sum_{j=1}^{p}{x}_{ij}$—and $\mathbf{P}$ captures the pattern effect (PE)—mean-centered deviations defining each person’s relative configuration.

Ipsatization (row-centering) produces ${\mathbf{X}}^{*}=\mathbf{X}-{\mathbf{x}}_{row}{1}_{p}^{{\top}}$, isolating pattern information and removing elevation. Applying SVD to ${\mathbf{X}}^{*}$ yields orthogonal component planes that summarize systematic within-person variation. Each two-dimensional plane (r=1,2,⋯) can be visualized as a *row-isometric biplot* (Gabriel, 1971) with persons as points and domains as vectors. The coordinates of persons ($\mathbf{F}$) and domain vectors ($\mathbf{B}$) form the analytic foundation for SEPA’s person-oriented outputs.

### Analytical innovations: Segment profiles and person-oriented indices

SEPA extends prior factor-analytic and biplot approaches by moving from purely visual interpretation to explicit, analyzable person-oriented quantities (see Supplementary Table S1). Within each retained plane r:

Across planes, SEPA performs variance-weighted aggregation using singular-value–based weights (${\alpha}_{r}\propto {\sigma}_{r}^{2}$) to form an overall segment profile while preserving plane-specific signal. No prior PAMS (e.g., Davison et al., 1996), FAPA (e.g., Kim, 2024), LPA (e.g., Asparouhov & Muthén, 2012), or classical biplot work (e.g., Gabriel, 1971) has defined this combination of plane-wise segment profiles, cosines, and plane-fit correlations as primary person-oriented indices for within-person inference.

**Box 1** Glossary of key SEPA terms.

**Table:** 

Term	Definition
Ipsatization	Row-centering of each person's scores across domains: ${x}_{ij}^{*}={x}_{ij}-{x}_{i}$. This removes each person's overall elevation so that the remaining variance reflects within-person profile shape (the pattern effect) exclusively
Level effect (LE)	Each person's mean score across all *p* domains: ${x}_{i\bullet }=\left(1/p\right)\sum_{j=1}^{p}{x}_{ij}$. It captures overall elevation, independent of profile shape
Pattern effect (PE)	The ipsatized residual after removing the level effect: $\mathbf{P}=\mathbf{X}-\mathbf{L}$. PE encodes the within-person configuration of relative strengths and weaknesses
Plane	A two-dimensional subspace defined by a pair of singular vectors (e.g., Dims 1–2 form Plane 1). Each plane represents one coherent pattern facet of within-person variation. Planes are orthogonal to one another and ordered by the proportion of pattern variance they explain jointly
Segment profile (${s}_{i}^{\left(r\right)}$)	For plane r, the vector of predicted ipsatized domain scores reconstructed from person i’s plane coordinates and domain loadings: ${\mathbf{s}}_{i}^{\left(r\right)}={\mathbf{F}}_{i}^{\left(r\right)}{\mathbf{B}}^{\left(r\right){\top}}$. Peaks indicate domains that are relative strengths for person i within that facet; valleys indicate relative weaknesses
Domain–person cosine (${c}_{ij}^{\left(r\right)}$)	The cosine of the angle between person i’s coordinate vector and domain j’s loading vector in plane r. Ranges from − 1 (perfect inverse alignment, i.e., strong relative weakness) to + 1 (perfect alignment, i.e., strong relative strength). Interpreted analogously to a correlation: |c|≥.71 ≈ ≥ 50% shared variance
Plane-fit index (${\rho}_{i}^{\left(r\right)}$)	The Pearson correlation between person i’s segment profile sᵢ(r) and the plane’s signed domain-length vector ${\mathbf{w}}^{\left(r\right)}$. Larger |ρ| indicates that this facet strongly characterizes the person’s profile shape; ρ near 0 indicates weak alignment with the plane’s structure. Rule of thumb: |ρ| ≥.70 = strong;.40 –.69 = moderate; <.40 = weak
Marker domain	A domain whose within-plane variance ${\Vert {b}_{j}^{(r)}\Vert }^{2}\ge 2/p$ (the average domain variance for a plane with total variance 2). Marker domains are the primary interpretive anchors for each plane
SV-weighted aggregation	Cross-plane combination of segment profiles (or cosines) using weights αᵣ proportional to each plane’s share of total pattern variance $\left({\alpha}_{r}\propto {d}_{\left(2r-1\right)}^{2}+{d}_{\left(2r\right)}^{2}\right)$. This preserves each facet’s relative contribution in the aggregated profile

*SVD* singular value decomposition; *LE* level effect; *PE* pattern effect. Notation follows the Method section. Equations are presented in simplified Unicode form; see the Method section for full typeset versions

### Relation to existing frameworks

- SEPA generalizes unidimensional SVD-based factor-analytic profile models to a genuinely plane-based system for multivariate behavioral data. It connects to—but extends beyond—the traditions of vector models (Carroll, 1972; Tucker, 1960), biplot geometry (Gabriel, 1971; Greenacre, 2010), and calibrated biplots (Gower & Hand, 1996; Gower et al., 2011; Graffelman & van Eeuwijk, 2005) by embedding a practical, reproducible workflow for person-centered applications. Specifically, SEPA adds: ipsatized SVD tailored to LE/PE separation;
- parallel analysis on the ipsatized matrix for selecting the PE space;
- bias-corrected and accelerated (BCa) bootstrap confidence intervals for plane stability;
- rule-based identification of marker domains ($\parallel {b}_{j}^{\left(r\right)}{\parallel }^{2}\ge 2/p$);
- subspace-stability diagnostics (principal angles and Procrustes comparisons) across ipsatization timing and samples.

Supplementary Table S1 summarizes these distinctions relative to Tucker/MDPREF, standard PCA biplots, and calibrated biplots, highlighting that only SEPA defines analyzable segment profiles, plane-wise person–domain cosines, and variance-weighted aggregation across multiple planes. Supplementary Table S2 contrasts SEPA with earlier single-axis profile analysis, emphasizing the shift from a unidimensional LE–PE axis to multi-plane, facet-specific profiles built on the same ipsatized SVD foundation.

### Why not a single-profile assignment?

Although PAMS, FAPA, and LPA produce richer person-level representations—correspondence weights or probability vectors that can be related to outcomes—these representations characterize patterns through a single underlying dimension or class. Users typically reduce them to a single best-fitting profile for interpretation. Either way, the representation does not directly articulate the variable contrasts that give profiles their shape. SEPA instead provides graded evidence across planes and domains: each individual’s degree of alignment with each domain within each facet, plus plane-fit indices that quantify how strongly each facet characterizes that person. This approach preserves multi-faceted individual differences and reveals trade-offs (e.g., high processing speed but low reasoning) that would be obscured by unidimensional or class-based profiling.

### Diagnostic and practical tools

Beyond defining new person-oriented outputs, SEPA offers empirical diagnostics for routine use:

- parallel analysis on the ipsatized matrix to determine the number of PE components;
- checks for LE dominance to verify that pattern variance is substantial;
- principal-angle and Procrustes tests to assess subspace equivalence across ipsatization timing or samples;
- plane-wise biplots with 95% BCa confidence intervals and cross-plane summary tables for reproducible reporting.

### Aims of the present study

This article pursues three aims:

1. *Define and formalize SEPA’s person-oriented outputs.* We articulate plane-wise segment profiles, domain–person cosines, plane-fit correlations, and variance-weighted aggregation across planes as core person-oriented indices for within-person profiling.
2. *Evaluate the empirical behavior of SEPA’s plane-wise summaries.* Using simulation, we examine the stability of segment profiles and cosines under varying sample sizes and variance structures, and illustrate how parallel analysis and subspace diagnostics guide selection of the PE space.
3. *Demonstrate applied value in multivariate cognitive assessment.* Using the Woodcock–Johnson IV cognitive battery, we show how SEPA identifies marker domains, reveals interpretable pattern facets across planes, and yields person-oriented indices that can be incorporated into standard regression models for clinical outcomes.

Collectively, these contributions position SEPA as a plane-wise, segment-based, and fully reproducible framework for modern person-centered analysis—uniting the geometric intuition of biplots with the person-oriented metrics needed for multivariate behavioral and clinical research.

## Method

### Data structure and preprocessing

Let $\mathbf{X}\in {\mathbb{R}}^{n\times p}$ contain observed domain scores (rows = persons i=1,⋯,n; columns = domains j=1,⋯,p). To emphasize pattern (within-person shape) rather than level, we ipsatize (row-center) the data to remove level effects for each person:

$${X}_{ij}^{*}={X}_{ij}-{X}_{i\bullet }, {X}_{i\bullet }=\frac{1}{p}\sum_{j=1}^{p}{X}_{ij}.$$

Then ${\sum}_{j=1}^{p}{X}_{ij}^{*}=0$ for all i, so the column space loses one degree of freedom and

$$\mathrm{r}\mathrm{a}\mathrm{n}\mathrm{k}\left({\mathbf{X}}^{*}\right)\le \mathrm{min}\left\{\mathrm{r}\mathrm{a}\mathrm{n}\mathrm{k}\left(\mathbf{X}\right),\hspace{0.17em}p-1\right\},$$

In particular, if n≥p and columns of $\mathbf{X}$ are linearly independent, then $\mathrm{r}\mathrm{a}\mathrm{n}\mathrm{k}({\mathbf{X}}^{*})=p-1$.

### Decomposition and planes

We apply singular-value decomposition to the ipsatized matrix,

$${\mathbf{X}}^{*}=\mathbf{U}\mathbf{D}{\mathbf{V}}^{\mathbf{\top }},$$

$\mathrm{w}\mathrm{i}\mathrm{t}\mathrm{h} \;\mathbf{D}=\mathrm{d}\mathrm{i}\mathrm{a}\mathrm{g}\left({d}_{1}, \dots , {d}_{K}\right), { d}_{1}\ge \cdots \ge {d}_{K}>0, \mathrm{a}\mathrm{n}\mathrm{d}\; K$ the number of retained singular components (via parallel analysis; see “Dimension Relevance”). We use a row-isometric biplot (Gabriel, 1971):

- Person coordinates (rows): $\mathbf{F}=\mathbf{U}\mathbf{D}$ (row i: ${\mathbf{F}}_{i}^{\mathbf{\top }}$).
- Domain vectors (columns): $\mathbf{B}=\mathbf{V}$ (row j: ${\mathbf{b}}_{j}^{\mathbf{\top }}$), anchored at the origin.

Axes are oriented so that the largest-magnitude domain loading on each dimension is positive; cosines inherit this orientation. Planes are defined as $\left(\mathrm{1,2}\right), \left(\mathrm{3,4}\right),$... with $P=\lfloor K/2\rfloor$. If K is odd, the last axis K is treated as a 1-D leftover axis.

### Projection rule and segment profiles

Within plane r with dimensions (a,b), define

$${\mathbf{F}}_{i}^{(r)}=\left({d}_{a}{u}_{ia},{d}_{b}{u}_{ib}\right), {\mathbf{b}}_{j}^{(r)}=\left({v}_{ja},{v}_{jb}\right).$$

The segment value for person i on domain j in plane r is the inner product

$${s}_{ij}^{(r)}={\mathbf{F}}_{i}^{(r)}{\mathbf{b}}_{j}^{(r)}={d}_{a}{u}_{ia}{v}_{ja}+{d}_{b}{u}_{ib}{v}_{jb}.$$

The segment profile is ${s}_{i}^{\left(r\right)}=({s}_{i1}^{\left(r\right)},\dots ,{s}_{ip}^{\left(r\right)}{)}^{{\top}}$. Peaks/valleys in ${s}_{i}^{\left(r\right)}$ indicate domain-specific strengths/weaknesses in that plane. Segment scores are on the ipsatized scale of ${\mathbf{X}}^{*}$.

### Two plane-wise summaries

- 1. Domain–person cosine (alignment)

$${c}_{ij}^{\left(r\right)}=cos\left(\angle \left({{\boldsymbol{F}}}_{i}^{\left(r\right)},{{\boldsymbol{b}}}_{j}^{\left(r\right)}\right)\right)=\frac{{{\boldsymbol{F}}}_{i}^{\left(r\right)}\bullet {{\boldsymbol{b}}}_{j}^{\left(r\right)}}{\Vert {{\boldsymbol{F}}}_{i}^{\left(r\right)}\Vert \Vert {{\boldsymbol{b}}}_{i}^{\left(r\right)}\Vert }\in \left[-\mathrm{1,1}\right]$$

quantifies how strongly person i aligns with domain j in plane r.

- 2. Domain–segment correlation (plane summary for a person)

Let the plane’s domain profile be the signed length of each domain vector, with sign taken from the larger-magnitude coordinate (ties broken by axis a):

$${w}_{j}^{(r)}=\mathrm{sign}\left({v}_{ja}\right)\sqrt{{v}_{ja}^{2}+{v}_{jb}^{2}}, {\mathbf{w}}^{(r)}=({w}_{1}^{(r)},\dots ,{w}_{p}^{(r)}{)}^{{\top}},$$

and *z*-score ${\mathbf{w}}^{(r)}$ across domains j (mean 0, SD 1). The plane-fit for person i is

$${\rho}_{i}^{(r)}=\mathrm{c}\mathrm{o}\mathrm{r}({{\boldsymbol{s}}}_{i}^{(r)},\hspace{0.17em} {{\boldsymbol{w}}}^{(r)}).$$

We refer to these as the domain–person cosin**e** ${c}_{ij}^{(r)}$ and the plane fit ${\rho}_{i}^{(r)}$.

### SV-weighted cross-plane aggregation

Define plane weights

$${\alpha}_{r}=\frac{{d}_{2r-1}^{2}+{d}_{2r}^{2}}{\sum_{k=1}^{K}{d}_{k}^{2}}, r=1,\dots ,\lfloor K/2\rfloor .$$

If K is odd, define the leftover-axis weight

$${\alpha}_{L}=\frac{{d}_{K}^{2}}{{\sum}_{k=1}^{K}{d}_{k}^{2}}.$$

For vector quantities (e.g., segment profiles), we aggregate across planes (and the leftover axis, if present):

$${s}_{i}=\sum_{r=1}^{\mathrm{P}}{s}_{i}^{(r)} \left(\mathrm{p}\mathrm{l}\mathrm{u}\mathrm{s}\; {s}_{i}^{\left(L\right)}\; \mathrm{i}\mathrm{f} \;K \;\mathrm{i}\mathrm{s} \;\mathrm{o}\mathrm{d}\mathrm{d}\right) .$$

For scalar aggregations (e.g., average cosines ${\overline{c} }_{ij}$, or averaged plane-fits ${\overline{\rho }}_{i}$), we treat a 1-D axis as half a plane by using modified weights:

$${\widetilde{\alpha }}_{r}={\alpha}_{r}, {\widetilde{\alpha }}_{L}=\frac{1}{2}{\alpha}_{L}, {\overline{c} }_{ij}=\frac{\sum_{r}{\widetilde{\alpha }}_{r}\hspace{0.17em}{c}_{ij}^{(r)}+{1}_{\{K odd\}}{\widetilde{\alpha }}_{L}\hspace{0.17em}{c}_{ij}^{(L)}}{\sum_{r}{\widetilde{\alpha }}_{r}+{1}_{\{K odd\}}{\widetilde{\alpha }}_{L}},$$

and analogously for ${\overline{\rho }}_{i}$.

#### Combining cosines across planes

Define the full vectors

$${\mathbf{F}}_{i}=\left({d}_{1}{u}_{i1},\dots ,{d}_{K}{u}_{iK}\right), {\mathbf{b}}_{j}=\left({v}_{j1},\dots ,{v}_{jK}\right).$$

The overall cosine between person i and domain j across *all* retained dimensions is

$${c}_{ij}^{(all)} = \frac{\sum_{r=1}^{P}{c}_{ij}^{(r)}\hspace{0.17em}\Vert {\mathbf{F}}_{i}^{\left(r\right)}\Vert \Vert {\mathbf{b}}_{j}^{\left(r\right)}\Vert }{\sqrt{\sum_{r=1}^{P}{\Vert {\mathbf{F}}_{i}^{\left(r\right)}\Vert }^{2}} \sqrt{\sum_{r=1}^{P}{\Vert {\mathbf{b}}_{j}^{\left(r\right)}\Vert }^{2}}} (adding\; the\; left\;over\; axis\; if\; K\; is\; odd) .$$

Thus, ${c}_{ij}^{(all)}$ is a length-weighted average of plane-wise cosines; opposite signs can cancel unless one plane dominates. We report plane-wise cosines in figures and tables and use ${c}_{ij}^{\left(\mathrm{a}\mathrm{l}\mathrm{l}\right)}$ as a concise summary when needed.

### Relation to R-/Q-type analysis

R-type factor analysis centers columns and models variable covariance; Q-type centers rows and models person covariance. Direct Q-type on the n×n person–person matrix is often infeasible. SEPA attains the Q-type perspective by applying SVD to row-centered ${\mathbf{X}}^{*}$ and interpreting row-metric biplots plane-wise—without forming the n×n matrix. Computationally, SVD on the n×p matrix ${\mathbf{X}}^{*}$ is $O(np\cdot \mathrm{m}\mathrm{i}\mathrm{n}\{n,p\})$: $O\left(n{p}^{2}\right)$ when n≥p and $O\left({n}^{2}p\right)$ when n<p.

### Dimension relevance

We determine the number of pattern components using Horn’s parallel analysis applied to ${\mathbf{X}}^{*}$ (Horn, 1965). In each replicate, we permute within columns, re-ipsatize rows, recompute the SVD, and compare observed ${d}_{k}^{2}$ values to 95th-percentile null thresholds. Column-wise permutation preserves each domain’s marginal distribution and missing-data pattern; re-ipsatization preserves row-centered “shape” under the null. Dimensions with ${d}_{k}^{2}$ above threshold are retained.

#### LE-masking check

To verify that pattern variance is not dominated by elevation, we compute the cosine between the first principal-component loadings from raw $\mathbf{X}$ and the constant vector ${1}_{p}/\sqrt{p}$. When this cosine is near 1 and the first component explains a large share of variance, elevation dominates and PE should be interpreted on ${\mathbf{X}}^{*}$.

### Implementation and reproducibility

We report R version, key package versions, and a global random-number seed. A single script reproduces all tables and figures from raw data, including ipsatization, SVD, plane construction, segment profiles, cosines, plane-fit indices, and cross-plane aggregations. Marker-domain confidence intervals are descriptive; when formal testing is desired within a plane, we recommend controlling the false discovery rate (Benjamini–Hochberg) at q=.05. For applied use, we recommend reporting n, p, preprocessing steps (ipsatization), retained K, plane weights ${\alpha}_{r}$, and the precise definition of ${w}^{\left(r\right)}$ used in ${\rho}_{i}^{\left(r\right)}$, along with code or supplementary files that compute ${s}_{i}^{\left(r\right)}$, ${c}_{ij}^{\left(r\right)}$, ${\rho}_{i}^{\left(r\right)}$, ${s}_{i}$, ${\overline{c} }_{ij}$, ${\overline{\rho }}_{i}$, and ${c}_{ij}^{\left(\mathrm{a}\mathrm{l}\mathrm{l}\right)}$. This R script implements the SEPA procedure, which is also available as a fully documented open-source R package on the Comprehensive R Archive Network (CRAN; https://cran.r-project.org/package=SEPA) and GitHub (https://github.com/sekangakim/sepa), as well as a Python implementation on the Python Package Index (PyPI; https://pypi.org/project/sepa-kim/).

### Simulation study

#### Objective

We conducted a Monte Carlo study to evaluate SEPA’s finite-sample behavior when the true LE/PE structure is known, focusing on (a) the accuracy of SEPA’s variance partitioning into level (LE) and pattern (PE) components, (b) the fidelity with which the estimated PE planes recover the generating pattern subspace, and (c) the stability of these estimates across different sample sizes and LE–PE variance configurations.

#### Design

Each dataset was simulated as an n×p matrix with n=200,500,1000 and p=7 domains, generated from

$$\mathbf{X}={{\boldsymbol{\upmu}}}_{LE}{1}_{p}^{{\top}}+\mathbf{Z}{{\boldsymbol{\Lambda}}}^{1/2}{\mathbf{B}}^{{\top}}+{\boldsymbol{\upvarepsilon}},$$

where ${{\boldsymbol{\upmu}}}_{\mathrm{LE}}\in {\mathbb{R}}^{n\times 1}$ contains person-specific elevations, ${1}_{p}$ is a p-vector of ones, $\mathbf{Z}\in {\mathbb{R}}^{n\times K}$ with ${Z}_{ik}\sim N\left(\mathrm{0,1}\right)$ and true K(=4), ${\boldsymbol{\Lambda}}={\mathrm{diag}}({\lambda}_{1},\dots ,{\lambda}_{K})$ specifies PE variances, $\mathbf{B}\in {\mathbb{R}}^{p\times K}$ is a fixed orthonormal loading matrix (${\mathbf{B}}^{{\top}}\mathbf{B}={I}_{K}$), and ${\boldsymbol{\upvarepsilon}}\sim N\left(0,{\sigma}_{\varepsilon }^{2}I\right)$ is independent residual noise. All components were mutually independent. We targeted total variance proportions of approximately 0.25: 0.65: 0.10 for LE, PE, and residual variance by choosing ${\sigma}_{LE}^{2}$, $\left\{{\lambda}_{k}\right\}$, and ${\sigma}_{\varepsilon }^{2}$ so that

$${\sigma}_{LE}^{2}+\sum_{k=1}^{K}{\lambda}_{k}+{\sigma}_{\varepsilon }^{2}=1.$$

One example parameter set was ${\sigma}_{LE}^{2}=0.25$, ${\sigma}_{\varepsilon }^{2}=0.10$, and Λ=diag(0.30,0.18,0.11,0.06). For each combination of n and ${\sigma}_{LE}^{2}\in \{0.10, 0.25, 0.40\}$, we generated 1000 replications using a single recorded seed; the loading matrix B was generated once (QR-orthonormalized Gaussian) and reused.

For each replicate, we ipsatized $\mathbf{X}$ to obtain ${\mathbf{X}}^{*}$, applied SVD, and fitted SEPA using the true K = 4 pattern components from the generating model. We then computed observed LE and PE variances from the generative decomposition X=L+P+ε and the corresponding latent variances from the SEPA estimates $\widehat{L}$ and $\widehat{P}$. Accuracy was summarized by

$${R}_{LE}^{2}=\frac{{V}_{LE, lat}}{{V}_{LE, obs}}, {R}_{PE}^{2}=\frac{{V}_{PE, lat}}{{V}_{PE, obs}},$$

where ${V}_{\_{\mathrm{obs}}}$ and ${V}_{\_{\mathrm{lat}}}$ are Frobenius-norm-based variance estimates per data entry. To evaluate the recovery of the PE pattern space, we computed Procrustes correlations ${R}_{\mathrm{Proc}}$ between the true loading matrix B and the estimated pattern loadings.

Across replications, we reported means, standard deviations, and 95% Monte Carlo confidence intervals for ${R}_{LE}^{2}$, ${R}_{PE}^{2}$, and ${R}_{Proc}$ under each condition, focusing on how performance changed with sample size and the magnitude of LE variance (Table 1).

**Table 1:** Monte Carlo evaluation of SEPA: Variance partitioning and subspace recovery (means over 1000 replications per condition)

*N*	LE Var ratio	${R}_{LE}^{2}$	${R}_{PE}^{2}$	${R}_{\mathrm{P}\mathrm{r}\mathrm{o}\mathrm{c}}$
200	0.10	1.00	0.93	0.97
500	0.10	1.00	0.93	0.97
1000	0.10	1.00	0.98	0.98
200	0.25	1.00	0.93	1.00
500	0.25	1.00	0.93	1.00
1000	0.25	1.00	0.93	1.00
200	0.40	1.00	0.93	1.00
500	0.40	1.00	0.93	1.00
1000	0.40	1.00	0.93	1.00

${R}_{LE}^{2}={V}_{LE, \mathrm{l}\mathrm{a}\mathrm{t}}/{V}_{LE, \mathrm{o}\mathrm{b}\mathrm{s}},$ where ${V}_{LE, \mathrm{l}\mathrm{a}\mathrm{t}}$ is the latent level-effect variance recovered by SEPA from the simulated data and ${V}_{LE, \mathrm{o}\mathrm{b}\mathrm{s}}$ is the observed level-effect variance computed directly from the raw simulated matrix; analogously, ${R}_{PE}^{2}={V}_{PE,\mathrm{l}\mathrm{a}\mathrm{t}}/{V}_{PE,\mathrm{o}\mathrm{b}\mathrm{s}}$ for pattern-effect variance. Both indices quantify how accurately SEPA recovers the generating LE and PE variance components. ${R}_{\mathrm{P}\mathrm{r}\mathrm{o}\mathrm{c}}$ is the Procrustes correlation between the estimated PE loading matrix and the true generating PE subspace. Values are means over 1000 replications per condition; 95% Monte Carlo confidence intervals appear in the Supplementary Materials

## Results

### Monte Carlo evaluation of SEPA

Across all sample sizes (*N* = 200, 500, 1000) and LE shares (10–40%), SEPA reproduced the observed level and pattern variance structure with negligible error. Latent and observed LE variances were numerically identical in every condition (${\mathrm{R}}_{LE}^{2}=1.00$), and PE variance recovery was very high (${\mathrm{R}}_{PE}^{2}=.93$–1.00). Subspace recovery was similarly strong: the estimated PE loading matrices exhibited high agreement with the true generating pattern subspace, with Procrustes correlations averaging from.97 to 1.00 across conditions. Recovery patterns were stable across *N* and LE proportions: ${R}_{\mathrm{P}\mathrm{r}\mathrm{o}\mathrm{c}}$ reached 1.00 for all LE ≥ 0.25 conditions and remained ≥.97 even at LE = 0.10, indicating robust finite-sample behavior for SEPA's ipsatized SVD framework.

### Empirical application of SEPA to real data

We applied SEPA to seven WJ-IV cognitive domains (or subscales) for n=5127 individuals (ages 10–70; M=24.37,SD=14.77; 52.4% female). Descriptive statistics appear in Table 2.

**Table 2:** Descriptive statistics of WJ-IV seven cognitive domain scores (*N* = 5127)

Domains	*M*	*SD*	Min	Max
LT	100.20	15.55	37.04	148.37
ST	100.93	15.72	35.77	159.30
CP	99.64	16.01	12.26	150.00
AP	101.01	15.61	36.55	151.35
VP	100.79	15.91	31.76	160.44
CK	100.92	15.75	38.34	153.93
FR	99.99	15.58	32.74	148.04

*LT* long-term retrieval; *ST* short-term working memory; *CP* cognitive processing speed; *AP* auditory processing; *VP* visual processing; *CK* comprehension knowledge; *FR* fluid reasoning. Values are on the natural scale prior to ipsatization

On the natural scale, PC1 showed strong alignment with the elevation vector, $\mathrm{c}\mathrm{o}\mathrm{s}\theta ({v}_{1},{1}_{p}/\sqrt{p})=0.998$, and accounted for 53.44% of total variance, consistent with LE dominance; hence we interpret PE on ${\mathbf{X}}^{\boldsymbol{*}}$ and report components from the ipsatized matrix ${\mathbf{X}}^{\boldsymbol{*}}$.

### Dimensionality and planes

SEPA was conducted on the ipsatized person × domain matrix (p=7), yielding rank p-1=6. Parallel analysis retained four dimensions explaining ~ 75% of pattern variance (dim1–dim4 = 23.6%, 21.5%, 15.4%, and 14.3%, respectively), which corresponded to eigenvalues of ${\mathbf{X}}^{*{\top}}{\mathbf{X}}^{*}$. We formed two orthogonal biplot planes:

- Plane 1: Dims 1–2 (= 45.09% total pattern variance)
- Plane 2: Dims 3–4 (= 29.68%), totaling 74.77% of the pattern variance.

Table 3 provides a high-level overview of what each plane captures and how cross-plane aggregation shifts individual profiles. Detailed domain coordinates, cosines, and segment profiles follow in the sections below.

**Table 3:** Plane summary overview for the WJ-IV SEPA application

Plane	Variance	Marker domains	Substantive contrast	Cross-plane shift
Plane 1	45.09% of PE variance	CP, VP	Processing speed (CP) vs. visual processing (VP). Individuals high on CP tend to be low on VP and vice versa within this facet	Dominant facet; combined profiles closely resemble Plane 1 characterizations $({\alpha}_{1} = 0.60$ 3)
Plane 2	29.68% of PE variance	CK, FR, ST	Comprehension-knowledge/fluid reasoning vs. short-term working memory. Individuals high on ST tend to be low on CK/FR within this facet	Adds ST-vs.-CK/FR contrast absent from Plane 1; shifts #944 and #1080 profiles toward ST in aggregated summaries $({\alpha}_{2} = 0.397)$
Aggregated	74.77% of PE variance (Planes 1 + 2)	-	Combined profiles (SV-weighted) preserve both contrasts. Plane 1 dominates; Plane 2 provides meaningful adjustments for individuals whose two-plane profiles diverge (e.g., #724 shows CP in Plane 1 but CK in Plane 2)	See Table 7 for overall cosines ${c}_{ij}^{\left(all\right)}$

*PE* pattern effect (ipsatized) variance. Marker domains exceed the within-plane threshold $\parallel {b}_{j}^{\left(r\right)}{\parallel }^{2} \ge 2/p=0.286$. Plane weights ${\alpha}_{1}$ and ${\alpha}_{2}$ are normalized to sum to 1. Dashes (-) indicate that the aggregated summary does not map to a single pair of marker domains

### Domain markers within planes

Within each plane, a domain’s contribution was summarized by its domain variance (squared coordinate length). Because each plane has total variance 2, the mean domain variance is 2/p (here 2/7=0.286). Domains with $\parallel {b}_{j}^{\left(r\right)}{\parallel }^{2} \ge 2/p$ are markers.

- Plane 1 (45.09%): CP (18%) and VP (11%) exceeded the 0.286 threshold → interpret this plane primarily as cognitive processing**/**visual processing.
- Plane 2 (29.68%): CK (8%), FR (6%), ST (5%) met/exceeded the threshold, with AP and VP near-threshold → interpret as knowledge/reasoning/memory (with AP/VP supporting).
- Across planes (“Sum across planes (%)” column): CP contributed most overall, followed by VP and CK (Table 4).

**Table 4:** Domain coordinates, 95% BCa CIs, length, domain variance, within plane %, and domain variance %

Plane 1 (Dims 1–2)—Domain contributions						
Domain	Dim 1 coordinate	Dim 2 coordinate	Length	Domain variance	Within-plane %	Domain variance %
LT	– 0.49 (–.53, –.44)	0.01	0.488	0.238	11.9	5.37
ST	0.33 (.29,.37)	– 0.19 (–.24, –.14)	0.380	0.144	7.22	3.26
CP	**0.45** (.43,.46)	**0.77** (.76,.79)	**0.895**	**0.801**	**40.05**	**18.06**
AP	0.15 (.10,.19)	– 0.31 (–.37, –.26)	0.348	0.121	6.06	2.73
VP	**– 0.64** (–.67, –.60)	**0.25** (.21,.30)	**0.687**	**0.472**	**23.63**	**10.65**
CK	0.15 (.10,.19)	– 0.43 (–.49, –.38)	0.458	0.210	10.5	4.73
FR	0.05	– 0.10 (–.16, –.04)	0.113	0.013	0.64	0.29
Dim Var. (%)	**23.6%**	**21.5%**			**100%**	**Plane 1: 45.09%**
Plane 2 (Dims 3–4)—Domain contributions						
Domain	Dim 3 coordinate	Dim 4 coordinate	Length	Domain variance	Within-plane %	Domain variance %
LT	– 0.45 (–.56, –.34)	0.11 (–.07,.27)	0.4624	0.214	10.69	3.17
ST	0.25 (.16,.34)	0.52 (.39,.65)	0.5825	0.339	16.96	5.04
CP	– 0.06	– 0.20 (–.26, –.13)	0.2076	0.043	2.16	0.64
AP	0.48 (.39,.59)	– 0.01	0.4886	0.239	11.94	3.54
VP	0.46 (.38,.53)	– 0.09	0.467	0.218	10.9	3.24
CK	**– 0.20** (–.25, –.14)	**– 0.71** (–.78, –.64)	**0.7446**	**0.554**	**27.72**	**8.23**
FR	**– 0.49** (–.59, –.38)	**0.38** (.22,.55)	**0.6265**	**0.392**	**19.62**	**5.82**
Dim Var. (%)	**15.4%**	**14.3%,**			**100%**	**Plane 2: 29.68%**
Summary across planes						
Domain	Sum across planes (%)					
AP	6.27					
CK	12.96					
CP	18.7					
FR	6.11					
LT	8.54					
ST	8.3					
VP	13.89					
Total %	74.77% (Plane 1 + Plane 2)					

The marker domain threshold =2/p (here 2/7=0.286); Domain variance ≥0.286 are treated as plane markers (bolded). CIs are 95% BCa bootstrap intervals (B = 5000). CIs spanning 0 are omitted to reduce clutter. Domain Variance % expresses each domain's contribution as a proportion of the total pattern variance across all retained dimensions (Planes 1 and 2 combined, accounting for 74.77% of the ipsatized matrix's total variance); it is not expressed as a proportion of total observed variance, which additionally includes level-effect and residual components

### Inter-domain and person–domain findings

#### How to read a SEPA plane and biplot

What a plane is. Each SEPA plane is a two-dimensional portrait of within-person profile variation. The two axes of the plane are singular vectors obtained from the ipsatized SVD; together, they define one coherent pattern facet. Domain arrows (vectors) and person points are projected onto this plane, allowing simultaneous visualization of which domains co-vary across persons and where each individual sits relative to those domains. Interpreting a plane always involves both axes jointly—neither axis is fully interpretable in isolation.

What person–domain alignment means. When a person's coordinate point projects in the direction of a domain's arrow—that is, when the angle between the person point and the domain arrow is small—the person's ipsatized score on that domain is above their personal mean: it is a relative strength within their profile. Conversely, when the person point lies in the opposite direction from a domain arrow, that domain is a relative weakness. The domain–person cosine ${c}_{ij}^{\left(r\right)}$ quantifies this alignment on a − 1 to + 1 scale. A cosine of.71 corresponds to approximately 50% shared variance (.71^2^ ≈.50), which we adopt as the threshold for 'strong' alignment (boldface in Tables 5 and 6). A cosine near 0 means the domain is neither a clear strength nor a clear weakness for that person within this facet.

**Table 5:** Domain–domain and person–domain cosines in Plane 1 (Dims 1–2) (header percentages are within-plane % contributions and sum to 100% within a plane)

Domain	LT (11.9%)	ST (7.2%)	CP (40.1%)	AP (6.1%)	VP (23.6%)	CK (11.0%)	FR (0.6%)
LT	1	**– 0.87**	– 0.48	– 0.44	**0.**9**4**	– 0.34	– 0.49
ST		1	0.00	**0.82**	– 0.99	**0.76**	**0.85**
CP			1	– 0.57	– 0.14	– 0.66	– 0.53
AP				1	**– 0.73**	**0.99**	**1.00**
VP					1	– 0.65	**– 0.77**
CK						1	**0.99**
FR							1
Person	LT	ST	**CP**	AP	**VP**	CK	FR
#724	**– 0.89** (–.95, – 0.84)	0.55 (.44,.68)	**0.83** (.79,.86)	– 0.01	– 0.66 (–.74, –.61)	– 0.12 (–.23, –.01)	0.04
#944	– 0.64 (–.72, –.56)	**0.93** (.88,.97)	– 0.36 (–.38, –.34)	**0.97** (.93, 1.00)	**– 0.87** (–.91, –.83)	**0.94** (.90,.97)	**0.98** (.81, 1.00)
#1080	0.25 (.13,.37)	0.25 (.10,.38)	**– 0.97** (–.98, –.95)	**0.76** (.65,.85)	– 0.11 (–.20, –.01)	**0.82** (.73,.90)	**0.72** (.25,.95)
#2117	0.62 (.44,.78)	**– 0.92** (–.98, –.83)	0.39 (.22,.53)	**– 0.98** (– 1.00, –.89)	**0.86** (.78,.92)	**– 0.95** (– 1.00, –.86)	**– 0.99** (– 1.00, –.88)

Entries are cosines (unitless, − 1 to 1) computed in Plane 1 (Dims 1–2). Column header list within-plane %, summing to 100% for the plane. Persons are #724, #944, #1080, #2117. We report bootstrap percentile 95% CIs (B = 5,000); CIs spanning 0 are omitted. Boldface indicates $\mid cosine\mid \ge .71$(≈ 50% shared variance)

**Table 6:** Domain–domain and person–domain cosines in Plane 2 (Dims 3–4) (header percentages are within-plane % contributions and sum to 100% within a plane)

Domain	LT (10.7%)	ST (17.0%)	CP (2.2%)	AP (11.9%)	VP (10.9%)	CK (27.7%)	FR (19.6%)
LT	1	– 0.21	0.05	**– 0.98**	**1.00**	0.03	**0.91**
ST		1	**– 0.99**	0.41	0.25	**– 0.98**	0.22
CP			1	– 0.26	– 0.10	**1.00**	– 0.37
AP				1	0.99	– 0.24	**– 0.80**
VP					1	– 0.08	**– 0.89**
CK						1	– 0.39
FR							1
Person	LT	ST	CP	AP	VP	CK	FR
#724	0.57	**−0.92** (– 1.00, –.46)	**0.85** (.28, 1.00)	**– 0.73** (–.95, –.42)	– 0.61 (–.99, –.08)	0.61 (.37,.99)	0.18
#944	– 0.50	**0.95**	**– 0.89**	0.66	0.53	– **0.89**	– 0.09
#1080	0.17	**0.93** (.84,.98)	**– 0.97** (– 1.00, –.84)	0.04	– 0.13	**– 0.98** (– 1.00, –.93)	0.57 (.28,.78)
#2117	**– 0.81** (–.98, –.51)	– 0.40 (–.56, –.21)	0.54 (.19,.80)	0.67 (.42,.88)	**0.78** (.59,.93)	0.56 (.42,.66)	**– 0.98** (– 1.00, –.94)

Entries are cosines (unitless; − 1 to 1) computed in Plane 2 (Dims 3–4). Column headers list within-plane % contributions, summing to 100% for the plane. Persons are #724, #944, #1080, #2117. We report bootstrap percentile 95% CIs (B = 5000); CIs spanning 0 are omitted. Boldface indicates $\mid cosine\mid \ge .71$

Four key biplot reading rules. (1) Opposite arrows = contrastive profile features. Domains whose arrows point in opposite directions (e.g., VP and ST in Plane 1) form a contrast: a person who peaks on VP tends to valley on ST, and vice versa. (2) Near-orthogonal arrows = independent profile features. Domains whose arrows are approximately perpendicular (e.g., CP and VP in Plane 1) are unrelated within this facet: knowing a person has a CP peak tells you nothing about whether they have a VP peak or valley. (3) Quadrant location = dominant domain cluster. A person point in a given quadrant projects positively onto the domain arrows pointing in that direction. For example, a person in the upper-right quadrant of Plane 1 has CP as a relative strength; a person in the lower-left projects opposite to CP, indicating a relative weakness. (4) Overlapping arrows = co-occurring profile features. Domains whose arrows nearly coincide (e.g., AP and CK in Plane 1; CP and CK in Plane 2) tend to peak and valley together across persons: a relative strength in one implies a relative strength in the other.

Why #724's segment profile is called a 'CP peak'. Person #724's coordinates in Plane 1 fall close to the direction of the CP domain arrow (cosine =.83, shared variance ≈ 69%). Person #724’s segment profile is the vector of predicted ipsatized domain scores reconstructed from #724's plane coordinates and the domain loadings. Because the projection is strongest toward CP, CP receives the highest predicted ipsatized score in this vector—hence the 'CP peak' label. The peak is not an assertion that CP is the objectively highest raw score; it means CP is the domain toward which #724's within-person pattern most strongly points within this facet.

#### Plane 1 biplot (Dims 1–2; 45.09%)

**Fig. 1:**
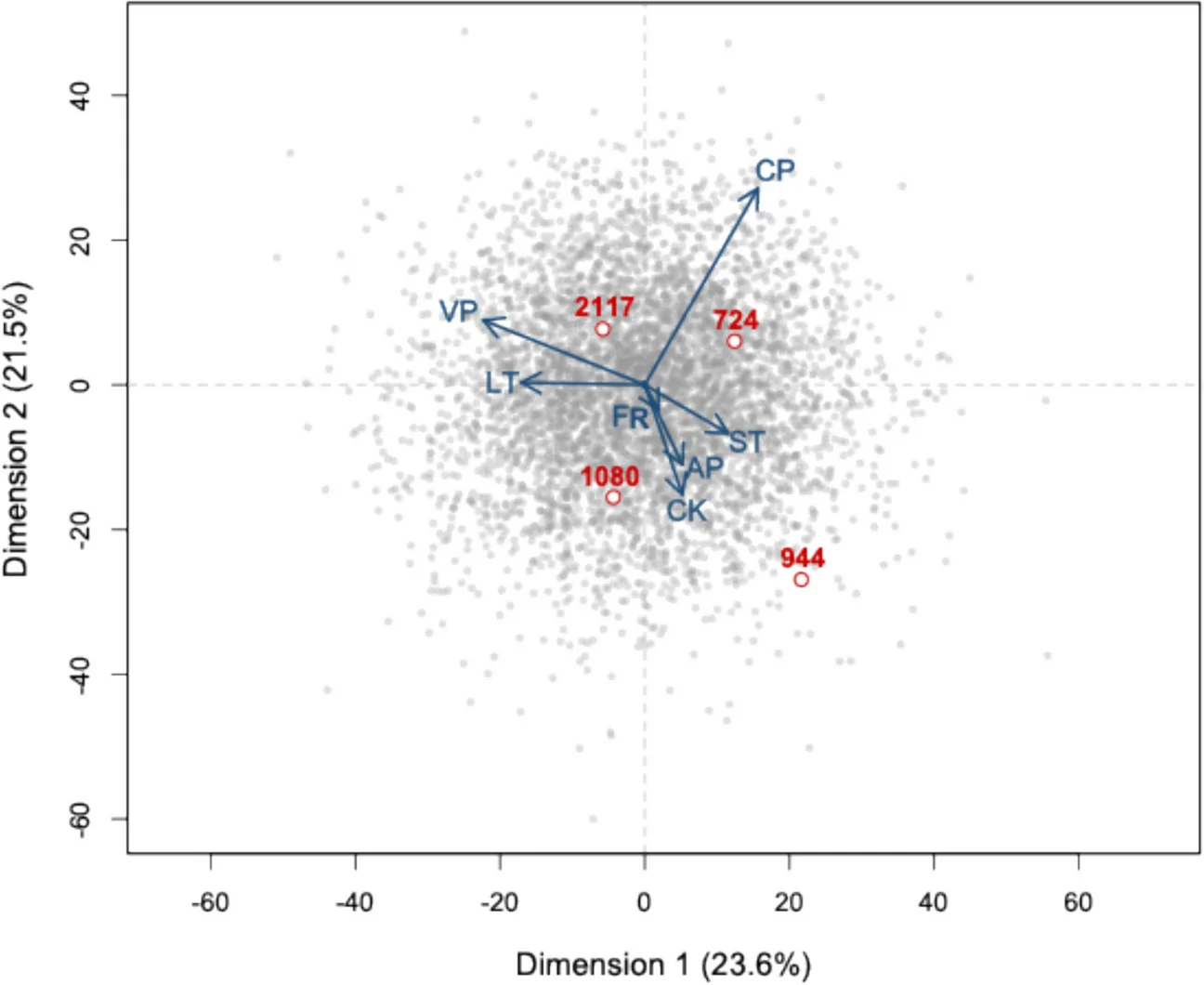
(Plane 1 biplot) domains and four pre-selected individuals projected onto Plane 1 (Dims 1–2). *Note.* Plane 1 biplot (Dims 1–2; 45.09% of PE variance). Domains are arrows; persons (#724, #944, #1080, #2117) are points. Arrow length indexes a domain’s within-plane variance; angles index cosines (associations). Small angles between a person and a domain arrow indicate large positive domain–person cosines; opposite directions indicate negative cosines. Marker domains (here CP, VP) have $\parallel {b}_{j}{\parallel }^{2} \ge 2/p$(threshold =2/7). Distances from the origin indicate within-plane profile differentiation ($\parallel {F}_{i}^{(1)}\parallel$)

To visually inspect associations in Plane 1, we present the biplot to complement the numerical estimates. Figure 1 shows the Plane 1 biplot (Dims 1–2; 45.09% of PE variance).

In Fig. 1, CP and VP are marker domains (longer arrows), whose variance ≥ the average variance of 2/7 = 0.2857 in Plane 1, defining the primary contrast on this plane. #724 projects strongly onto CP (arrow-parallel), #944 projects toward {ST, AP, CK, FR}, #1080 projects toward {FR, AP**,** CK**}**, and #2117 projects toward VP. Distances from the origin quantify profile differentiation; #944 is farthest (most differentiated) from the origin, compared to the others. Small angles between a person point and a domain arrow correspond to large positive domain–person cosines. The numerical summary of the cosine angles between domains and between individuals and domains is reported in Table 5.

In Table 5, rows for #724, #944, #1080, #2117 show person–domain cosines and the cosines in bold indicate $|\mathrm{c}\mathrm{o}\mathrm{s}\mathrm{i}\mathrm{n}\mathrm{e}|\ge .71$ (≈ 50% shared variance, since.${71}^{2}\approx .50$). The 95% bootstrap percentile CIs (B = 5000) are shown beneath cosine estimates, where CIs that include 0 are omitted for readability (absence of a CI indicates it spans zero). Negative values indicate alignment in the opposite direction along the plane.

From now on, we use the symbol *r* for cosines in biplot planes and explicitly write ‘Pearson *r*’ when reporting correlations of observed variables. The following are summaries for inter-domain and person-domain cosines from Table 5.

Angles among domain vectors reflect correlations:

- Strong positive (small angle): {LT, VP} and {ST, AP, CK, FR} (e.g., r=.76-.94)
- Near-zero/negative with others: CP is roughly orthogonal/obtuse to most non-visual domains
- Three clusters appeared: {CP}, {ST, AP, CK, FR}, {LT, VP}

We illustrate SEPA with four individuals who share the same raw mean (= 100) but occupy different positions, along with different person-domain alignments in Plane 1:

- #724: aligns with CP (e.g., r=.83); segment profile shows a CP peak.
- #944: aligns with ST/AP/CK (r s ≥.93); segment profile peaks on those domains.
- #1080: aligns with AP/CK (r s =.76–.83); segment profile peaks on AP and CK.
- #2117: aligns with VP (r=.86); segment profile shows a VP peak.

##### Profile differentiation

Plane 1 (Dims 1–2)**.** Distance from the origin is indexed by the Euclidean norm of the person scores in Plane 1, $\parallel {\mathbf{F}}_{i}^{(1)}\parallel =\sqrt{({d}_{1}{u}_{i1}{)}^{2}+({d}_{2}{u}_{i2}{)}^{2}}$; larger values indicate a more differentiated (peaky) profile within Plane 1. Values (Fig. 1): #724 = 12.08**,** #944 = 30.23**,** #1080 = 14.11**,** #2117 = 8.44. Thus #944 is farthest (greatest within-plane pattern variance, since $\parallel {\mathbf{F}}_{i}^{\left(1\right)}{\parallel }^{2}$ is the variance contribution, whereas #2117 is closest (flattest).

#### Plane 2 biplot (Dims 3–4; 29.68%)

**Fig. 2:**
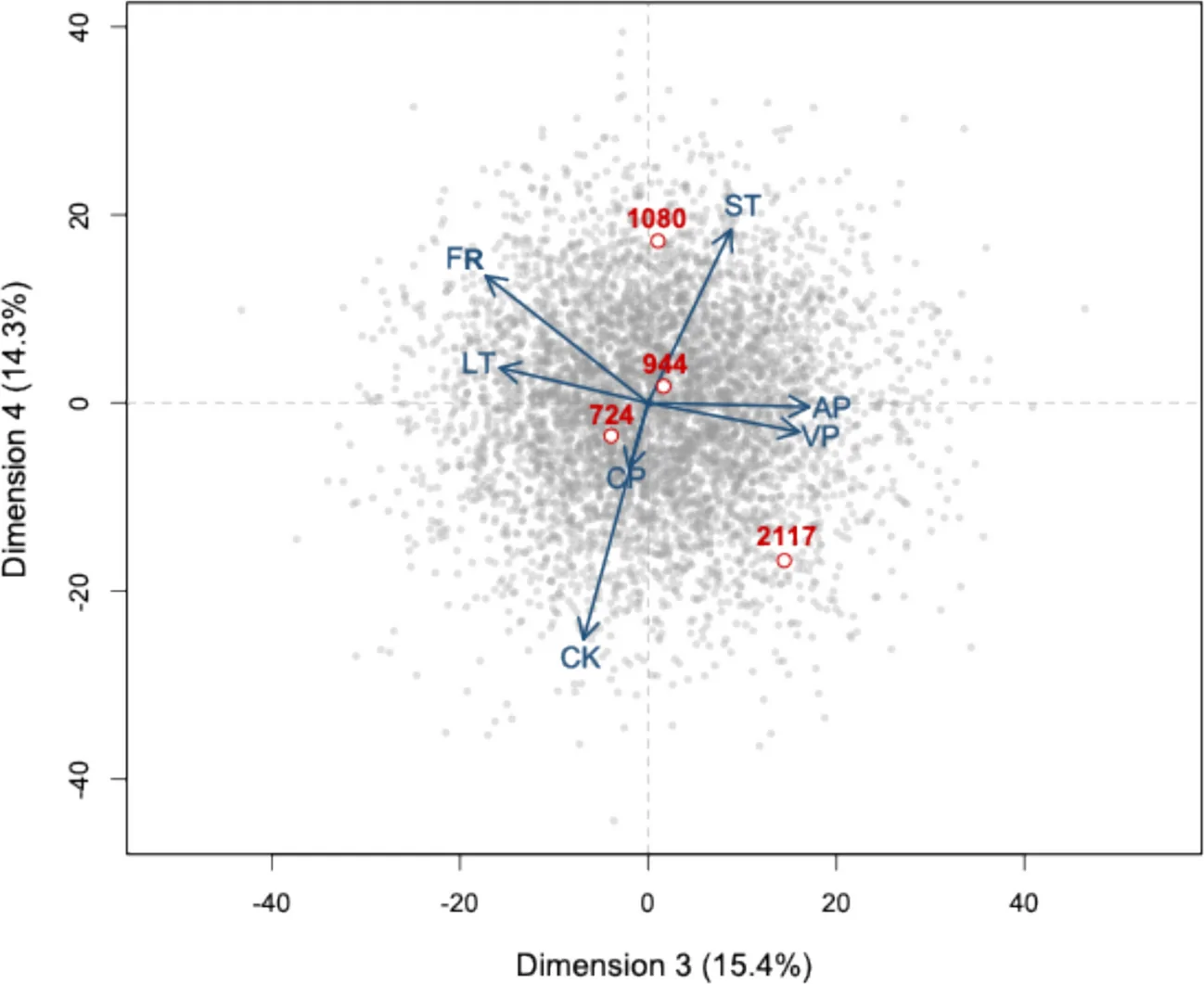
(Plane 2 biplot). Domains and four pre-selected individuals projected onto Plane 2 (Dims 3–4). *Note.* Plane 2 biplot (Dims 3–4; 29.68% of PE variance). Notable domain relations: CP–CK collinear, LT–AP nearly opposite, AP–VP strongly aligned, ST opposed to {CP, CK}. Person projections show #944 and #1080 aligned with ST, #724 toward CK, #2117 toward VP/AP. This plane adds an ST vs. CK/FR contrast not captured by Plane 1; SV-weighted aggregation shifts combined profiles accordingly

Plane 1 revealed a processing speed vs. visual processing contrast as the dominant pattern facet. Plane 2 adds a substantively different story: a comprehension-knowledge/fluid reasoning vs. short-term working memory contrast that is entirely absent from Plane 1. Individuals who appeared similar in Plane 1 (e.g., #944 and #1080, both aligning away from CP) diverge clearly in Plane 2, with both now aligning strongly toward ST—a shift that only becomes visible when the second plane is examined. Figure 2 shows the Plane 2 biplot (Dims 3–4; 29.68% of PE variance).

Plane 2 adds 29.68% of PE variance. Notable domain relations (Table 6, upper panel): CP–CK collinear (r=1.00); LT–AP nearly opposite (r=-.98); AP–VP strongly positive (r=.99); ST strongly negative with {CP, CK} (r=-.99,-.98). Person projections show #944 and #1080 aligning with ST (r=.95,.93), #724 toward CK (r=.85), and #2117 toward VP/AP (r=.78,.67). This plane introduces the ST vs. CK/FR contrast absent from Plane 1; SV-weighted aggregation therefore shifts combined profiles for #944 and #1080 toward ST.

In Table 6, rows for **#**724, #944, #1080, #2117 show person–domain cosines and the cosines in bold indicate |cosine|≥.71 (≈ 50% shared variance, since.71^2^ ≈.50). The 95% bootstrap percentile CIs (B = 5,000) are shown beneath cosine estimates, where CIs that include 0 are omitted for readability (absence of a CI indicates it spans zero). Negative values indicate alignment in the opposite direction along the plane.

The following are summaries for inter-domain and person-domain cosines from Table 6:

Angles among domain vectors reflect correlations:

Plane 2 adds 29.68% of pattern variance. Notable domain relations (in the upper part of Table 6):

- CP–CK perfectly collinear (r=1.00);
- LT–AP nearly opposite (r=-0.98);
- AP–VP strongly positive (r=.99);
- ST–{CP, CK**}** strongly negative (r=-.99,-.98).

We illustrate SEPA with four individuals who share the same raw mean (= 100) but occupy different positions, along with different person-domain alignments in Plane 2:

- #944 and #1080 show strong alignment with ST (r =.95,.93). *For #944 the 95% bootstrap CI includes 0, so interpret cautiously despite the large magnitude (shared variance* =.95.^2^** = **.90*)*
- #724 aligns with CK (r=.85);
- #2117 shows moderate alignment with AP and VP (r=.67,.78).

**Profile differentiation**—Plane 2 (Dims 3–4). The same norm in Plane 2, $\parallel {\mathbf{F}}_{i}^{(2)}\parallel =\sqrt{({d}_{3}{u}_{i3}{)}^{2}+({d}_{4}{u}_{i4}{)}^{2}}$, yields (Fig. 2): #724 = 4.61, #944 = 2.11, #1080 = 15.10, #2117 = 19.38. Here #2117 is farthest (most differentiated), while #944 is nearest the origin (flatter).

### Segment profiles

Segment profiles are the predicted domain values from projections, ${s}_{ij}^{(r)}=\langle {\mathbf{F}}_{i}^{(r)},{\mathbf{b}}_{j}^{(r)}\rangle$, in the ipsatized scale (mean-centered per person). (Full segment-profile tables are available in the Supplementary Materials.) Larger $\mid {s}_{ij}^{(r)}\mid$ indicates stronger within-plane alignment; the sign indicates direction.

As expected from the cosines (see Tables 5 and 6):

- #724: CP peak on Plane 1.
- #944: ST/AP/CK peaks on Plane 1.
- #1080: AP/CK peaks on Plane 1, with ST peak on Plane 2.
- #2117: VP peak on Plane 1, with AP/VP elevation on Plane 2.

The raw ipsatized scores underlying these biplot positions are provided in Supplementary Table S3. Because no single plane accounts for all pattern variance, peaks in observed ipsatized profiles may be most strongly expressed in different planes across individuals: the peaks of #724 (CP, + 8.56), #944 (ST, + 17.10), and #2117 (VP, + 9.56) are most strongly captured in Plane 1, whereas the peak of #1080 (ST, + 12.54) is most strongly captured in Plane 2 (cosine =.93 vs..25 in Plane 1). All valleys in the observed ipsatized profiles are recoverable in either Plane 1 or Plane 2. This cross-plane pattern confirms that SEPA's cosine structure faithfully reflects each individual's observed within-person profile across the retained planes jointly.

Note that when a domain’s within-plane contribution is near zero (very short arrow; e.g., FR on Plane 1), even a large cosine can be unstable; interpretation should defer to arrow length (domain variance) in that plane.

### Aggregated summaries across planes

We formed combined segment profiles by aggregating Plane 1 and Plane 2 contributions (Figs. 1–2):

$${\mathbf{S}}_{i}={\alpha}_{1}\hspace{0.17em}{\mathbf{s}}_{i}^{(1)}+{\alpha}_{2}\hspace{0.17em}{\mathbf{s}}_{i}^{(2)},$$

where Plane 1 and Plane 2 account for 45.09% and 29.68% of pattern variance, yielding normalized weights ${\alpha}_{1}=0.603,\text{ \hspace{0.05em}}{\alpha}_{2}=0.397 \left({\alpha}_{1}+{\alpha}_{2}=1\right)$.

Overall cosines ${c}_{ij}^{(\mathrm{a}\mathrm{l}\mathrm{l})}$(Dims 1–4) provide a concise summary consistent with the SV-weighted segment profiles (see Table 7); values near ±1 indicate strong alignment across planes, whereas small magnitudes often reflect proximity to the origin in at least one plane (hence wider CIs).

**Table 7:** Overall cosines for four individuals across Planes 1 and 2 and 95% bootstrap percentile CIs

Person	LT	ST	CP	AP	VP	CK	FR
#724	– 0.46 (–.56, –.40)	0.01	0.82 (.77,.85)	– 0.22 (–.34, –.09)	– 0.64 (–.72, –.52)	0.20 (.01,.33)	0.07
#944	– 0.49 (–.55, –.44)	0.56 (.50,.65)	– 0.37 (–.39, –.34)	0.60 (.47,.72)	– 0.70 (–.76, –.62)	0.44 (.40,.50)	0.17 (.09,.26)
#1080	0.21 (.03,.37)	0.66 (.56,.72)	– 0.81 (–.88, –.74)	0.32 (.13,.51)	– 0.11 (–.21, –.01)	– 0.31 (–.38, –.16)	0.50 (.30,.64)
#2117	– 0.33 (–.46, –.12)	– 0.51 (–.63, –.36)	0.26 (.23,.29)	0.27 (.07,.44)	0.69 (.57,.79)	0.24 (.11,.30)	– 0.96 (–.97, –.91)

${c}_{ij}^{(\mathrm{a}\mathrm{l}\mathrm{l})}$ are overall person–domain cosines aggregated across Planes 1 and 2 (Dims 1–4) as a length-weighted average of plane-wise cosines; values near ±1 indicate strong alignment across planes. CIs are bootstrap percentile 95% (B = 5000); CIs spanning 0 are omitted

Because Plane 1 explains a larger share, combined profiles resemble Plane 1 while systematically adjusted by Plane 2:

- #724: strengthened CP.
- #944: marginally dominant AP/ST/CK pattern.
- #1080: elevation on ST/FR with lower CP.
- #2117: strongest on VP, with lower FR.

(${c}_{ij}^{(\mathrm{a}\mathrm{l}\mathrm{l})}$ in Table 7 provides a concise cross-plane cosine; it is the length-weighted average of plane-wise cosines and aligns with these aggregated profiles.)

### Plane fit (ρ): Definition and interpretation

For each person *i* and plane *r*, we quantify how well the person’s segment profile aligns with the plane’s domain pattern using

$${\rho}_{i}^{(r)}=\mathrm{c}\mathrm{o}\mathrm{r}({s}_{i}^{(r)},\hspace{0.17em}{w}^{(r)}),$$

where ${s}_{i}^{(r)}={F}_{i}^{(r)}{{B}^{(r)}}^{{\top}}$ is the person’s reconstructed domain profile (referred to as a segment profile) in plane r (from the person coordinates ${F}_{i}^{(r)}$ and domain loadings ${B}^{(r)}$), and ${w}^{(r)}$ is the *z*-standardized, signed domain-length vector for that plane (sign taken from the first axis; magnitude $\parallel {b}_{j}^{(r)}\parallel$). Larger ρ indicates stronger alignment with the plane’s pattern; negative ρ indicates an inverse pattern; values near 0 indicate weak or no alignment. The results are for Plane 1 and Plane 2 as follows:

- Plane 1** (**${\rho }^{(1)}$**)**: ${\rho}_{724}^{(1)}=0.92$, ${\rho}_{944}^{(1)}=0.54$, ${\rho}_{1080}^{(1)}=-0.35$, ${\rho}_{2117}^{(1)}=-0.52$: Person **#**724 shows an excellent fit to Plane 1’s domain pattern; #944 shows a moderate fit. Persons #1080 and #2117 exhibit inverse patterns relative to Plane 1 (moderate and stronger inversion, respectively).
- Plane 2 (${\rho }^{(2)}$): ${\rho}_{724}^{(2)}=-0.94$, ${\rho}_{944}^{(2)}=0.91$, ${\rho}_{1080}^{(2)}=0.44$, ${\rho}_{2117}^{(2)}=0.30$: Person #724 aligns strongly but inversely with Plane 2, whereas #944 aligns strongly positively with Plane 2. Persons #1080 and #2117 show weaker positive alignment.

*Rule-of-thumb*: $\mid \rho \mid \ge .70$≈ strong alignment; $.40\le \mid \rho \mid <.70$≈ moderate; $\mid \rho \mid <.40$≈ weak.

## Discussion

### Overview and main contributions

Segmented profile analysis (SEPA) reframes profile analysis as a plane-wise decomposition of within-person variance grounded in ipsatized SVD. Rather than assigning individuals to a single latent profile, SEPA models multiple interpretable planes that capture distinct pattern facets and then aggregates them using singular-value weights. In the WJ-IV illustration, parallel analysis yielded four pattern components forming two planes (Plane 1: 45.09%; Plane 2: 29.68%; total: 74.77% of PE variance).

The Monte Carlo study showed that SEPA accurately recovers the generating variance structure and pattern space: latent LE and PE variances closely matched their observed counterparts $\left({R}_{LE}^{2}\approx 1.00, {R}_{PE}^{2}\approx .93-1.00\right)$, and the estimated PE loading matrices exhibited high Procrustes agreement with the true pattern subspace (≈.97–1.00 across conditions). These results support the use of ipsatized SVD as a stable basis for plane-wise profiling in practical sample sizes.

Empirically, the WJ-IV planes were substantively interpretable: Plane 1 was dominated by cognitive processing speed and visual processing, whereas Plane 2 highlighted a comprehension/fluency versus working-memory contrast. SEPA’s person-oriented quantities—segment profiles, domain–person cosines, and plane-fit indices—summarized how individual patterns align with these facets.

### What SEPA adds to profile analysis

SEPA contributes three elements beyond unidimensional and class-based approaches:

1. *Plane-wise person profiles*
  For each plane r, the segment profile ${s}_{i}^{\left(r\right)}={F}_{i}^{\left(r\right)}{{B}^{\left(r\right)}}^{{\top}}$ represents person *i*’s projected pattern across domains, while domain–person cosines ${c}_{ij}^{\left(r\right)}$ and the plane-fit index ${\rho}_{i}^{\left(r\right)}$ quantify alignment with that facet. These are treated as person-oriented indices with clear variance properties rather than purely visual aids.
2. *Principled cross-plane aggregation*
  Planes are combined via singular-value weights ${\alpha}_{r}$, yielding aggregated profiles ${S}_{i}={\sum}_{r}{\alpha}_{r}{s}_{i}^{\left(r\right)}$ and overall cosines ${c}_{ij}^{\left(all\right)}$. This preserves the contribution of each facet instead of collapsing everything into a single axis. In the WJ-IV data, Plane 2 added a knowledge/fluency vs. working-memory contrast that shifts combined profiles for some individuals, clarifying why superficially similar raw means mask different pattern structures.
3. * Diagnostics for multidimensional heterogeneity*
  Cross-plane discordance—opposite-signed cosines for the same domain across planes—indicates that the domain behaves differently by facet. SEPA therefore highlights multidimensional complexity that single-profile and latent-class approaches can obscure, especially when interpretation is restricted to marker domains $\left({\Vert {b}_{j}^{\left(r\right)}\Vert }^{2}={b}_{ja}^{2}+ {b}_{jb}^{2}\ge 2/p\right)$ with large $\left|C_{ij}^{(r)}\right|$.

#### Substantive and clinical implications

SEPA characterizes individuals by graded alignment with multiple facets rather than by membership in a single latent class. In cognitive or clinical applications, this supports:

- *Facet-matched hypotheses:* plane-fit indices (ρ) and domain–person cosines can be used as predictors of facet-specific outcomes (e.g., speed vs. reasoning), rather than a single “type” score.
- *Treatment matching:* among marker domains, large $\left|{c}_{ij}^{(r)}\right|$ and $\left|{\rho}_{i}^{(r)}\right|$ identify individuals whose patterns strongly conform to a given facet, suggesting priority targets for intervention.
- *Change monitoring:* longitudinal changes in ${c}_{ij}^{\left(r\right)}$, ${\rho}_{i}^{\left(r\right)}$, and distances from the origin $\Vert {F}_{i}^{(r)}\Vert$ provide plane-wise indices of improvement, deterioration, or emerging heterogeneity. Reliable-change thresholds for these quantities could be developed via bootstrap or test–retest designs.

#### Practical reporting guidelines

To support cumulative use of SEPA, we recommend reporting:

- retained K and plane variances;
- marker domains per plane and summaries of ρ and c (e.g., medians, IQRs, thresholds such as |c|≥.70 as “strong”);
- plane biplots with representative persons and BCa CIs for domain coordinates;
- machine-readable tables of segment profiles, plane-wise cosines, overall cosines, and plane-fit indices;
- analytic details (ipsatization, parallel-analysis settings, orientation/sign rules, missing-data handling, RNG seed, and code).

### Methodological considerations and future directions

SEPA operates on the ipsatized (pattern) scale, with LE handled separately; it is intended to complement, not replace, classical factor models and mixtures. Orientation constraints and linear geometry should be checked, and SV-weighted aggregates treated as summaries rather than substitutes for plane-wise inference. Future work could examine regularized or sparse variants for high-p settings, longitudinal SEPA in which planes evolve over time, and ROC-based clinical cut-points for ρ and c anchored to external outcomes.

## Conclusion

SEPA provides a plane-wise, person-centered framework for profiling multidomain assessments. The simulation study demonstrated accurate recovery of level/pattern variance and pattern subspaces, and the WJ-IV application illustrated how SEPA’s segment profiles, cosines, and plane-fit indices yield reproducible, interpretable summaries for assessment, treatment matching, and monitoring change.

## Supplementary Information

Below is the link to the electronic supplementary material.

Supplementary file1 (PDF 248 kb)

## Data Availability

The data analyzed in this study were derived from the Woodcock–Johnson IV Tests of Cognitive Abilities norming sample (Schrank et al., 2014). These data are proprietary and owned by Riverside Insights and therefore cannot be shared publicly. To support reproducibility, we provide a synthetic dataset (*wj4_fake.csv*) in the Supplementary Materials that approximates the observed marginal distributions and the qualitative LE/PE structure assumed by SEPA. The synthetic data were generated from an additive model incorporating a dominant person-oriented elevation component, a four-dimensional orthonormal pattern component, and residual noise, and were calibrated to match the reported means and standard deviations. All analyses are fully reproducible using the synthetic data and the annotated R scripts provided in the Supplementary Materials. Access to the original WJ-IV data is available from Riverside Insights under their standard licensing agreements (https://www.riversideinsights.com).

## Code availability

All R scripts used for the analyses are included in the Supplementary Materials. SEPA is already publicly available as an open-source R package on CRAN (https://cran.r-project.org/package=SEPA) and GitHub (https://github.com/sekangakim/sepa), with a Python implementation on PyPI (https://pypi.org/project/sepa-kim/).

The complete R script used in the present study is included in the supplementary material and data are available upon request.

## Declarations

### Open practices statement

Analysis code is provided in the Supplementary Materials, submitted alongside the manuscript. The data set used in this study is proprietary but may be made available upon reasonable request. None of the analyses were preregistered. Additionally, SEPA is freely available as an open-source R package on the Comprehensive R Archive Network (CRAN; https://cran.r-project.org/package=SEPA) and on GitHub (https://github.com/sekangakim/sepa), and as a Python implementation on the Python Package Index (PyPI; https://pypi.org/project/sepa-kim/).
