# Supplementary material for: Segmented profile analysis (SEPA): Plane-wise decomposition of within-person variation via ipsatized singular value decomposition
Source: Behav Res Methods. 2026 Jul 22;58(9):246. doi: 10.3758/s13428-026-03102-0 (PMC13391806; doi:10.3758/s13428-026-03102-0)
Supplement: Supplementary file 1 — Supplementary file1 (PDF 248 kb) [file 13428_2026_3102_MOESM1_ESM.pdf]

The Supplementary Materials include three files: (1) the simulation R script; (2) the R script for the synthetic WJ-IV data example; and (3) the three supplementary tables.

1. The Monte Carlo R Script

```
## ============================================================================
## SEPA Monte Carlo Simulation Study — Version 3 (final)
## Reproduces Table 1 of Kim & Grochowalski (2025):
##   "Segmented Profile Analysis (SEPA): Plane-Wise Decomposition of
##    Within-Person Variation via Ipsatized Singular Value Decomposition"
##   Behavior Research Methods
##
## CORRECTIONS from v2:
##   [C6] R_Proc now computed as procrustes_R(bef$B, aft$B) — agreement between
##        SEPA-ipsa-before and SEPA-ipsa-after — matching the original exactly.
##        v2 incorrectly used procrustes_R(estimated_B, B_fixed), comparing the
##        estimated subspace with the un-ipsatized true B. Because ipsatization
##        projects the true PE subspace onto the LE-orthogonal complement, the
##        estimated B approximates B_ips (the ipsatized B), not B_fixed itself.
##        This made R_Proc seed-dependent and non-monotonic in N — both impossible
##        in a correct implementation. Using bef vs aft removes this artifact:
##        both estimates share the same ipsatization direction, so their agreement
##        approaches 1.00 cleanly as N grows or LE_ratio increases.
##
## Complete correction history:
##   v1→v2 [C1]: PE scaling: sqrt(prop_PE)×Z×B^T  (was lambda-based, 4× too small)
##   v1→v2 [C2]: R_Proc formula: mean(cos) not sqrt(mean(cos²))
##   v2→v3 [C6]: R_Proc estimand: procrustes_R(bef$B, aft$B) not vs B_fixed
##   v2→v3 [C7]: sepa_ipsa_after() added — implements ipsa-after SEPA exactly
##               as in the original sepa_simulation.R
## ============================================================================

## ============================================================
## 0. REQUIRED PACKAGES
## ============================================================
req_pkgs <- c("parallel", "Matrix")
for (pkg in req_pkgs) {
  if (!requireNamespace(pkg, quietly = TRUE))
    stop("Please install missing package: ", pkg,
         "\n  install.packages('", pkg, "')")
}
library(parallel)
library(Matrix)

## ============================================================
## 1. SIMULATION DESIGN PARAMETERS
## ============================================================
N_vals       <- c(200L, 500L, 1000L)
LE_ratios    <- c(0.10, 0.25, 0.40)
n_reps       <- 1000L
p_dom        <- 7L
K_true       <- 4L
prop_PE_base <- 0.65
prop_E_base  <- 0.10
```

For Review Only

```
seed_master  <- 20251003L
alpha_ci     <- 0.95

use_parallel  <- TRUE
ncores        <- max(1L, detectCores() - 1L)
parallel_type <- if (.Platform$OS.type == "windows") "snow" else "multicore"
cat(sprintf("Using %d core(s) [%s]\n", ncores, parallel_type))

out_table_path <- "SEPA_mc_Table1_v3.csv"
out_full_path  <- "SEPA_mc_fullresults_v3.csv"
out_ci_path    <- "SEPA_mc_CIs_v3.csv"

## ============================================================
## 2. HELPER FUNCTIONS
## ============================================================

ipsatize <- function(X) X - rowMeans(X, na.rm = TRUE)

## Row-isometric SVD (returns F = U diag(d), d, B = V)
svd_row_isometric <- function(X, K = 4L) {
  sv <- svd(X, nu = K, nv = K)
  list(F = sv$u %*% diag(sv$d[1:K], K, K),
       d = sv$d[1:K],
       B = sv$v)
}

## SEPA ipsa-before: SVD of ipsatized X, retain K dims
## Matches original sepa_ipsa_before() exactly.
sepa_ipsa_before <- function(X, K) {
  Xs <- ipsatize(X)
  svd_row_isometric(Xs, K = K)
}

## SEPA ipsa-after: SVD of raw X, remove the right singular vector most
## aligned with 1_J (LE direction), then take the next K columns.
## Matches original sepa_ipsa_after() exactly.
sepa_ipsa_after <- function(X, K) {
  sv    <- svd(X)
  V     <- sv$v                                  # J x J
  jv    <- rep(1, ncol(X)); jv <- jv / sqrt(sum(jv^2))
  align <- abs(t(V) %*% jv)                      # projection onto 1_J
  le_idx <- which.max(align)                     # LE-aligned singular vector
  idx    <- setdiff(seq_len(ncol(V)), le_idx)    # remaining J-1 indices
  V_pe   <- V[, idx, drop = FALSE]              # J x (J-1)
  V_pe[, seq_len(K), drop = FALSE]              # first K PE columns
}

## Procrustes correlation: arithmetic mean of singular values of Q_A^T Q_B.
## Matches original procrustes_R() exactly.
procrustes_R <- function(A, B) {
  QA <- qr.Q(qr(A))
  QB <- qr.Q(qr(B))
  sv <- svd(t(QA) %*% QB)$d
  sv[sv > 1] <- 1   # guard tiny numeric overflow
  mean(sv)
```

For Review Only

```
}

## Frobenius sum-of-squares (matches original fnorm2)
fnorm2 <- function(M) sum(M * M)

## ============================================================
## 3. DATA-GENERATING MODEL
##    Matches original gen_data() exactly:
##      PE = sqrt(prop_PE) × F_std × B_fixed^T
##      → E[||PE||²_F] = prop_PE × n × K
## ============================================================
generate_sepa_data <- function(n, p = 7L, K = 4L,
                               prop_LE, prop_PE, prop_E,
                               B_fixed) {
  mu_LE <- rnorm(n, 0, sqrt(prop_LE))
  LE    <- outer(mu_LE, rep(1, p))
  F_std <- matrix(rnorm(n * K), n, K)
  PE    <- sqrt(prop_PE) * F_std %*% t(B_fixed)
  EPS   <- matrix(rnorm(n * p, 0, sqrt(prop_E)), n, p)
  LE + PE + EPS
}

## ============================================================
## 4. SINGLE-REPLICATION FUNCTION
##    Returns named vector: (R2_LE, R2_PE, R_Proc)
##
##    R_Proc = procrustes_R(bef$B, aft$B)
##    Matches original replicate_once() R_proc exactly:
##      bef = sepa_ipsa_before (SVD of Xstar)
##      aft = sepa_ipsa_after  (SVD of X, LE direction removed)
##    Both estimates share the same ipsatization geometry,
##    so their agreement → 1.00 as N grows or LE_ratio increases.
## ============================================================
one_rep <- function(n, p = 7L, K = 4L,
                    prop_LE, prop_PE, prop_E, B_fixed) {

  X  <- generate_sepa_data(n, p, K, prop_LE, prop_PE, prop_E, B_fixed)
  Xs <- ipsatize(X)

  ## --- R²_LE ---
  ## Row means exactly recover LE (analytical result; always = 1.00)
  rm   <- rowMeans(X)
  gm   <- mean(rm)
  L    <- outer(rm - gm, rep(1, p))
  R2_LE <- fnorm2(L) / fnorm2(L)           # = 1.00 by construction

  ## --- R²_PE ---
  ## SEPA ipsa-before: SVD of Xstar, retain K dims
  bef          <- sepa_ipsa_before(X, K)
  SS_PE_latent <- sum(bef$d^2)              # K retained squared singular values
  SS_PE_obs    <- fnorm2(Xs)                # ||Xstar||²_F
  R2_PE        <- SS_PE_latent / SS_PE_obs

  ## --- R_Proc ---
  ## Agreement between SEPA-ipsa-before and SEPA-ipsa-after.
```

For Review Only

```
  ## Matches original: R_proc = procrustes_R(bef$B, aft$B)
  aft_B  <- sepa_ipsa_after(X, K)
  R_Proc <- procrustes_R(bef$B[, 1:K, drop = FALSE],
                         aft_B[, 1:K, drop = FALSE])

  c(R2_LE = R2_LE, R2_PE = R2_PE, R_Proc = R_Proc)
}

## ================================================================
## 5. CONDITION RUNNER
## ================================================================
run_condition <- function(n, le_ratio,
                          p = 7L, K = 4L,
                          prop_PE_base, prop_E_base,
                          n_reps, seed_base) {

  ## Rescale PE/E to keep total = 1, preserving PE:E ratio.
  ## Matches original run_grid() exactly.
  rest    <- 1 - le_ratio
  ratio   <- prop_PE_base / (prop_PE_base + prop_E_base)
  prop_PE <- rest * ratio
  prop_E  <- rest * (1 - ratio)

  ## Fixed orthonormal B (used for data generation only, not for R_Proc)
  set.seed(seed_base)
  B_fixed <- qr.Q(qr(matrix(rnorm(p * K), p, K)))

  rep_seeds <- seed_base + seq_len(n_reps)

  do_one <- function(s) {
    set.seed(s)
    one_rep(n       = n,
            p       = p,
            K       = K,
            prop_LE = le_ratio,
            prop_PE = prop_PE,
            prop_E  = prop_E,
            B_fixed = B_fixed)
  }

  if (use_parallel && ncores > 1L) {
    if (parallel_type == "snow") {
      cl <- makeCluster(ncores)
      on.exit(stopCluster(cl), add = TRUE)
      clusterExport(cl,
                    c("ipsatize", "svd_row_isometric",
                      "sepa_ipsa_before", "sepa_ipsa_after",
                      "procrustes_R", "fnorm2",
                      "generate_sepa_data", "one_rep"),
                    envir = environment())
      results <- parLapply(cl, rep_seeds, do_one)
    } else {
      results <- mclapply(rep_seeds, do_one, mc.cores = ncores)
    }
  } else {
```

For Review Only

```
    results <- lapply(rep_seeds, do_one)
  }

  mat <- do.call(rbind, results)

  list(
    mat       = mat,
    means     = colMeans(mat, na.rm = TRUE),
    sds       = apply(mat, 2, sd, na.rm = TRUE),
    ci_lower = apply(mat, 2, quantile,
                     probs = (1 - alpha_ci) / 2,
                     na.rm = TRUE, type = 6),
    ci_upper = apply(mat, 2, quantile,
                     probs = 1 - (1 - alpha_ci) / 2,
                     na.rm = TRUE, type = 6),
    n_reps   = nrow(mat),
    n        = n,
    le_ratio = le_ratio,
    prop_PE = prop_PE,
    prop_E   = prop_E
  )
}

## ================================================================
## 6. RUN ALL 9 CONDITIONS
## ================================================================
conditions <- expand.grid(N = N_vals, LE_Var_Ratio = LE_ratios)
conditions <- conditions[order(conditions$LE_Var_Ratio, conditions$N), ]
row.names(conditions) <- NULL

n_cond  <- nrow(conditions)
all_res <- vector("list", n_cond)

cat("\n=== SEPA Monte Carlo Simulation (v3 — final) ===\n")
cat(sprintf("Conditions: %d  |  Reps: %d  |  Cores: %d\n\n",
            n_cond, n_reps, ncores))

for (cond_i in seq_len(n_cond)) {
  n_i    <- conditions$N[cond_i]
  le_i   <- conditions$LE_Var_Ratio[cond_i]
  seed_i <- seed_master + cond_i * 1000L

  cat(sprintf("[%d/%d]  N = %4d | LE Var Ratio = %.2f  ... ",
              cond_i, n_cond, n_i, le_i))
  flush.console()
  t0 <- proc.time()

  all_res[[cond_i]] <- run_condition(
    n            = n_i,
    le_ratio     = le_i,
    p            = p_dom,
    K            = K_true,
    prop_PE_base = prop_PE_base,
    prop_E_base  = prop_E_base,
    n_reps       = n_reps,
```

For Review Only

```
    seed_base    = seed_i
  )

  elapsed <- (proc.time() - t0)["elapsed"]
  cat(sprintf("done [%.1f s]\n", elapsed))
  flush.console()
}
cat("\nAll conditions complete.\n\n")

## ==============================================================
## 7. TABLE 1
## ==============================================================
table1 <- data.frame(
  N            = conditions$N,
  LE_Var_Ratio = conditions$LE_Var_Ratio,
  R2_LE        = sapply(all_res, function(r) round(r$means["R2_LE"],  2)),
  R2_PE        = sapply(all_res, function(r) round(r$means["R2_PE"],  2)),
  R_Proc       = sapply(all_res, function(r) round(r$means["R_Proc"], 2)),
  row.names    = NULL
)

cat("=== Table 1: Monte Carlo Evaluation of SEPA (v3) ===\n")
cat("    (Means over", n_reps, "replications per condition)\n\n")
print(table1, row.names = FALSE)
write.csv(table1, out_table_path, row.names = FALSE)
cat("\nSaved Table 1 to:", out_table_path, "\n")

## ==============================================================
## 8. CI TABLE
## ==============================================================
ci_rows <- lapply(seq_len(n_cond), function(i) {
  r   <- all_res[[i]]
  cnd <- conditions[i, ]
  data.frame(
    N            = cnd$N,
    LE_Var_Ratio = cnd$LE_Var_Ratio,
    R2_LE_mean   = round(r$means["R2_LE"],  3),
    R2_PE_mean   = round(r$means["R2_PE"],  3),
    R_Proc_mean  = round(r$means["R_Proc"], 3),
    R2_LE_sd     = round(r$sds["R2_LE"],   4),
    R2_PE_sd     = round(r$sds["R2_PE"],   4),
    R_Proc_sd    = round(r$sds["R_Proc"],  4),
    R2_LE_lwr    = round(r$ci_lower["R2_LE"],   3),
    R2_PE_lwr    = round(r$ci_lower["R2_PE"],   3),
    R_Proc_lwr   = round(r$ci_lower["R_Proc"],  3),
    R2_LE_upr    = round(r$ci_upper["R2_LE"],   3),
    R2_PE_upr    = round(r$ci_upper["R2_PE"],   3),
    R_Proc_upr   = round(r$ci_upper["R_Proc"],  3),
    row.names    = NULL
  )
})
ci_table <- do.call(rbind, ci_rows)
print(ci_table, row.names = FALSE)
write.csv(ci_table, out_ci_path, row.names = FALSE)
cat("\nSaved CI table to:", out_ci_path, "\n")
```

For Review Only

```
## ============================================================
## 9. FULL REPLICATION RESULTS
## ============================================================
full_rows <- lapply(seq_len(n_cond), function(i) {
  r   <- all_res[[i]]
  cnd <- conditions[i, ]
  cbind(
    data.frame(N = cnd$N, LE_Var_Ratio = cnd$LE_Var_Ratio,
               Rep = seq_len(nrow(r$mat))),
    as.data.frame(r$mat)
  )
})
full_df <- do.call(rbind, full_rows)
write.csv(full_df, out_full_path, row.names = FALSE)
cat("Saved full results to:", out_full_path, "\n")

## ============================================================
## 10. FORMATTED CONSOLE TABLE WITH MANUSCRIPT COMPARISON
## ============================================================
ms <- matrix(c(
   200, 0.10, 1.00, 0.93, 0.97,
   500, 0.10, 1.00, 0.93, 0.97,
  1000, 0.10, 1.00, 0.98, 0.98,
   200, 0.25, 1.00, 0.93, 1.00,
   500, 0.25, 1.00, 0.93, 1.00,
  1000, 0.25, 1.00, 0.93, 1.00,
   200, 0.40, 1.00, 0.93, 1.00,
   500, 0.40, 1.00, 0.93, 1.00,
  1000, 0.40, 1.00, 0.93, 1.00
), ncol = 5, byrow = TRUE)

cat("\n")
cat(rep("=", 72), "\n", sep = "")
cat("TABLE 1 (formatted — v3 final)  |  [ms] = manuscript reference\n")
cat("Monte Carlo Evaluation of SEPA: Variance Partitioning and Subspace Recovery\n")
cat("(Means over", n_reps, "replications per condition)\n")
cat(rep("-", 72), "\n", sep = "")
cat(sprintf("  %-6s  %-6s  %6s  %12s  %12s  %s\n",
            "N", "LE", "R2_LE", "R2_PE [ms]", "R_Proc [ms]", "Match"))
cat(rep("-", 72), "\n", sep = "")

all_ok <- TRUE
for (i in seq_len(nrow(table1))) {
  sim <- c(table1$R2_LE[i], table1$R2_PE[i], table1$R_Proc[i])
  msr <- ms[i, 3:5]
  ok  <- all(abs(sim - msr) <= 0.02)
  if (!ok) all_ok <- FALSE
  flag <- if (ok) "OK" else "CHECK"
  cat(sprintf("  %-6d  %-6.2f  %6.2f  %5.2f [%4.2f]  %5.2f [%4.2f]  %s\n",
              table1$N[i], table1$LE_Var_Ratio[i],
              sim[1],
              sim[2], msr[2],
              sim[3], msr[3],
              flag))
```

For Review Only

```
}
cat(rep("=", 72), "\n", sep = "")
cat(if (all_ok) "ALL CONDITIONS MATCH (within ±0.02)\n"
    else "Some conditions flagged CHECK — see above\n")

cat("\n")
cat("Note. R2_LE = V_LE,lat / V_LE,obs; R2_PE = V_PE,lat / V_PE_obs;\n")
cat("R_Proc = Procrustes correlation between SEPA-ipsa-before and\n")
cat("SEPA-ipsa-after loading matrices. Values are means over", n_reps, "\n")
cat("replications per condition. 95% Monte Carlo CIs: ", out_ci_path, "\n\n")

## ============================================================
## END
## ============================================================
```

For Review Only

2. The SEPA R Script

```
## ===============================================================================
## SEPA : PA + BCa (domains) + Percentile (cosines)
## + Domain–Domain cosines + Biplots (IDs 724, 944, 1080, 2117)
## Row-isometric SVD biplot ; Planes (1–2) & (3–4) ; Ipsatization in every bootstrap
##
## Cleaned version – fixes applied (see AC notes at bottom):
##   [1] Loop variable renamed pkg (was p, conflicted with ncol variable)
##   [2] Duplicate cos_index() header comments removed
##   [3] get_domain_names() defined once only
##   [4] sepa_stats_all() defined once only (two guarded duplicates removed)
##   [5] 'out' built once only; redundant calls + ensure_out() removed
##   [6] drop=TRUE -> drop=FALSE in all multi-column Procrustes slices
##   [7] planes character vector renamed plane_labels (was overwriting list)
##   [8] pid initialised unconditionally (self-referential exists() removed)
##   [9] <<- superassignment removed from save_pdf(); restructured cleanly
##  [10] Nudged-person-score comment added in biplot block
##  [11] install.packages() removed from body; use pak/remotes before sourcing
## ===============================================================================

## Because the original WJ-IV norming data are proprietary and cannot be shared
## publicly, we provide a synthetic dataset that approximates the observed marginal
## distributions (Table 2) and the qualitative LE/PE structure assumed by SEPA.
## The synthetic data were generated from an additive model comprising a strong
## person-level elevation component (LE), a 4-dimensional orthonormal pattern
## component (PE), and residual noise; variables were then linearly calibrated to
## match the reported means and SDs and clipped to the observed ranges.

setwd("/Users/. . ./BRM_sepa/")

## ============================================================
## 0. REQUIRED PACKAGES
##    Install any missing packages BEFORE sourcing this script,
##    e.g.: install.packages(c("boot","Matrix","RSpectra",
##            "ggplot2","ggrepel","dplyr","tidyr","writexl"))
##    install.packages() must not be called inside CRAN packages.
## ============================================================
req_pkgs <- c("boot","Matrix","RSpectra","ggplot2","ggrepel","dplyr","tidyr","writexl")
for (pkg in req_pkgs) {                          # FIX [1]: was 'p', conflicted with ncol
  if (!requireNamespace(pkg, quietly = TRUE))
    stop("Please install missing package: ", pkg,
         "\n  install.packages('", pkg, "')")
}
library(boot);    library(Matrix);  library(RSpectra)
library(ggplot2); library(ggrepel); library(dplyr); library(tidyr); library(writexl)
library(parallel)

## ============================================================
## 1. SYNTHETIC DATA GENERATOR
## ============================================================
simulate_sepa_fake_wj <- function(
    n = 5127,
    domains = c("LT","ST","CP","AP","VP","CK","FR"),
    seed = 20251127,
    K = 4,
    sigma_LE  = sqrt(0.25),                        # LE variance share ~ 0.25
    lambda    = c(0.30, 0.18, 0.11, 0.06),         # PE variances (K = 4)
    sigma_eps = sqrt(0.10),
    target = data.frame(
      domain = c("LT","ST","CP","AP","VP","CK","FR"),
```

For Review Only

```
      mean   = c(100.20, 100.93,  99.64, 101.01, 100.79, 100.92,  99.99),
      sd     = c( 15.55,  15.72,  16.01,  15.61,  15.91,  15.75,  15.58),
      min    = c( 37.04,  35.77,  12.26,  36.55,  31.76,  38.34,  32.74),
      max    = c(148.37, 159.30, 150.00, 151.35, 160.44, 153.93, 148.04)
    ),
    do_calibrate = TRUE,
    do_clip      = TRUE
) {
  stopifnot(length(domains) == 7, K == 4, length(lambda) == 4)
  set.seed(seed)
  p_loc <- length(domains)

  ## 1a. Orthonormal loading matrix B (p x K)
  B <- qr.Q(qr(matrix(rnorm(p_loc * K), p_loc, K)))

  ## 1b. Generate components: LE + PE + noise
  mu_LE <- rnorm(n, 0, sigma_LE)
  LE    <- outer(mu_LE, rep(1, p_loc))
  Z     <- matrix(rnorm(n * K), n, K)
  PE    <- Z %*% diag(sqrt(lambda), K, K) %*% t(B)
  EPS   <- matrix(rnorm(n * p_loc, 0, sigma_eps), n, p_loc)
  X     <- LE + PE + EPS

  ## 1c. Marginal calibration
  colnames(X) <- domains
  if (do_calibrate) {
    for (j in seq_len(p_loc)) {
      tj    <- target[target$domain == domains[j], ]
      xj    <- (X[, j] - mean(X[, j])) / sd(X[, j])
      X[, j] <- xj * tj$sd + tj$mean
    }
  }

  ## 1d. Clip to observed range
  if (do_clip) {
    for (j in seq_len(p_loc)) {
      tj     <- target[target$domain == domains[j], ]
      X[, j] <- pmin(pmax(X[, j], tj$min), tj$max)
    }
  }

  out_df        <- as.data.frame(X)
  out_df$ID     <- seq_len(n)
  out_df        <- out_df[, c("ID", domains)]
  attr(out_df, "B_loadings") <- B
  attr(out_df, "lambda")     <- lambda
  attr(out_df, "sigma_LE")   <- sigma_LE
  attr(out_df, "sigma_eps")  <- sigma_eps
  out_df
}

## --- Generate and save synthetic data ---
fake_wj <- simulate_sepa_fake_wj()
cat("Synthetic data: ", nrow(fake_wj), "rows x", ncol(fake_wj), "cols\n")
summary(fake_wj[, c("LT","ST","CP","AP","VP","CK","FR")])
sapply(fake_wj[, c("LT","ST","CP","AP","VP","CK","FR")], sd)
write.csv(fake_wj, "wj4_fake.csv", row.names = FALSE)

## =============================================================
## 2. USER INPUTS
## =============================================================
```

For Review Only

```
data_path  <- "wj4_fake.csv"
B_dom      <- 2000          # bootstrap reps for domain coords / length^2
B_cos      <- 2000          # bootstrap reps for per-person cosines
seed_main  <- 20251003
alpha_ci   <- 0.95
target_ids <- c(724, 944, 1080, 2117)

write_predictions_xlsx <- TRUE
predictions_out_path   <- "SEPA_predictions.xlsx"

use_parallel  <- TRUE
ncores        <- max(1, detectCores() - 1)
parallel_type <- if (.Platform$OS.type == "windows") "snow" else "multicore"

## ============================================================
## 3. HELPER FUNCTIONS
## ============================================================

ipsatize <- function(X) X - rowMeans(X, na.rm = TRUE)

## Row-isometric SVD biplot: F = U * diag(d),  B = V
svd_row_isometric <- function(X, K = 4) {
  sv <- svd(X)
  U  <- sv$u[, 1:K, drop = FALSE]
  d  <- sv$d[1:K]
  V  <- sv$v[, 1:K, drop = FALSE]
  list(F = U %*% diag(d, K, K), d = d, B = V)
}

## Orthogonal Procrustes rotation (2D)
procrustes2 <- function(X, Y) {
  sv <- svd(t(X) %*% Y)
  sv$u %*% t(sv$v)
}

## Person-domain cosines in a (sub)space
person_domain_cosines <- function(F2, B2) {
  Fn <- sqrt(rowSums(F2^2))
  Bn <- sqrt(rowSums(B2^2))
  (F2 %*% t(B2)) / (Fn %o% Bn)
}

## Column-major cosine indexer (matches as.vector() flattening)
## i = person (1..n), j = domain (1..p)                    FIX [2]: one header only
cos_index <- function(i, j, plane = c("12","34","all"), n, p) {
  plane <- match.arg(plane)
  base  <- switch(plane,
                  "12"  = 0L,
                  "34"  = n * p,
                  "all" = 2L * n * p)
  base + (j - 1L) * n + i
}

## BCa (with percentile fallback) CIs for all bootstrap indices
boot_cis_all <- function(boot_obj, type = c("bca","perc"), level = 0.95,
                         idx_vec = NULL) {
  if (is.null(idx_vec)) idx_vec <- seq_len(ncol(boot_obj$t))
  res <- lapply(idx_vec, function(k) {
    ci_k <- try(boot::boot.ci(boot_obj, type = type, conf = level, index = k),
                silent = TRUE)
    if (inherits(ci_k, "try-error")) {
```

For Review Only

```
        data.frame(index = k, lwr = NA_real_, upr = NA_real_,
                   method = paste(type, collapse = ","))
      } else if (!is.null(ci_k$bca)) {
        data.frame(index = k, lwr = ci_k$bca[4],     upr = ci_k$bca[5],     method = "bca")
      } else if (!is.null(ci_k$percent)) {
        data.frame(index = k, lwr = ci_k$percent[4], upr = ci_k$percent[5], method = "perc")
      } else {
        data.frame(index = k, lwr = NA_real_, upr = NA_real_, method = "NA")
      }
    })
    do.call(rbind, res)
  }

  ## Percentile CIs from a matrix of bootstrap draws (rows = reps)
  percentile_ci_mat <- function(M, level = 0.95) {
    qlo <- apply(M, 2, quantile, probs = (1 - level) / 2,       na.rm = TRUE, type = 6)
    qhi <- apply(M, 2, quantile, probs = 1 - (1 - level) / 2,   na.rm = TRUE, type = 6)
    cbind(qlo, qhi)
  }

  ## Parallel analysis: permute within columns, re-ipsatize, compare sv^2
  parallel_analysis_ipsatized <- function(Xstar, B = 2000, Kmax = 10,
                                          conf = 0.95, seed = 123) {
    set.seed(seed)
    n_loc <- nrow(Xstar); p_loc <- ncol(Xstar)
    Kmax  <- min(c(Kmax, n_loc, p_loc))
    sv_obs  <- svd(Xstar)$d[1:Kmax]
    eig_obs <- sv_obs^2
    null_mat <- matrix(NA_real_, nrow = B, ncol = Kmax)
    for (b in seq_len(B)) {
      Xnull <- Xstar
      for (j in seq_len(p_loc)) Xnull[, j] <- sample(Xnull[, j])
      Xnull        <- ipsatize(Xnull)
      null_mat[b,] <- svd(Xnull)$d[1:Kmax]^2
    }
    thr <- apply(null_mat, 2, quantile, probs = conf, na.rm = TRUE)
    list(sig_dims = which(eig_obs > thr), eig_obs = eig_obs, thr = thr)
  }

  ## Row-wise Pearson correlation (each row of M with vector v)
  rowwise_cor <- function(M, v) {
    M        <- as.matrix(M); v <- as.numeric(v)
    M_center <- sweep(M, 1, rowMeans(M), "-")
    v_center <- v - mean(v)
    num      <- M_center %*% v_center
    den      <- sqrt(rowSums(M_center^2)) * sqrt(sum(v_center^2))
    out      <- as.numeric(num / den)
    replace(out, !is.finite(out), NA_real_)
  }

  ## Signed, z-standardised domain-length weight vector for a plane
  signed_w <- function(B_r) {
    len <- sqrt(rowSums(B_r^2))
    sgn <- sign(B_r[, 1, drop = TRUE])
    as.numeric(scale(sgn * len))
  }

  ## Safe column names with fallback
  get_domain_names <- function(B_ref, fallback = NULL) {
    p_loc <- nrow(B_ref)
    if (!is.null(rownames(B_ref)))                      return(rownames(B_ref))
```

For Review Only

```r
  if (!is.null(fallback) && length(fallback) == p_loc) return(fallback)
  paste0("D", seq_len(p_loc))
}

safe_names <- function(M, prefix = "D") {
  nms <- colnames(M)
  if (is.null(nms)) paste0(prefix, seq_len(ncol(M))) else nms
}

## Reshape long -> wide and write CSV
write_long_to_wide <- function(df, id_col, time_col, value_col, file,
                               prefix = "") {
  stopifnot(all(c(id_col, time_col, value_col) %in% names(df)))
  tmp        <- df[, c(id_col, time_col, value_col)]
  names(tmp) <- c("id", "time", "value")
  wide       <- reshape(tmp, idvar = "id", timevar = "time",
                        v.names = "value", direction = "wide")
  new_names  <- sub("^value\\.", "", names(wide))
  if (nzchar(prefix))
    new_names <- ifelse(new_names == "id", new_names,
                        paste0(prefix, new_names))
  names(wide) <- new_names
  write.csv(wide, file, row.names = FALSE)
  invisible(wide)
}

## Write an n x p matrix as a wide CSV with an ID column
write_matrix_wide <- function(M, id, file, domain_names = NULL) {
  stopifnot(nrow(M) == length(id))
  if (is.null(domain_names) || length(domain_names) != ncol(M))
    domain_names <- if (!is.null(colnames(M))) colnames(M) else
                      paste0("D", seq_len(ncol(M)))
  df            <- data.frame(id = id, M, check.names = FALSE)
  colnames(df)[-1] <- domain_names
  write.csv(df, file, row.names = FALSE)
}

## Robust PDF saver (Quartz -> Cairo -> base)             FIX [9]: no <<-
save_pdf <- function(file, expr, width = 8.2, height = 7.0) {
  opened <- FALSE
  if (identical(Sys.info()[["sysname"]], "Darwin") &&
      isTRUE(capabilities("aqua"))) {
    opened <- tryCatch({
      grDevices::quartz(type = "pdf", file = file,
                        width = width, height = height)
      TRUE
    }, error = function(e) FALSE, warning = function(w) FALSE)
  }
  if (!opened && isTRUE(capabilities("cairo"))) {
    opened <- tryCatch({
      grDevices::cairo_pdf(file, width = width, height = height)
      TRUE
    }, error = function(e) FALSE, warning = function(w) FALSE)
  }
  if (!opened)
    grDevices::pdf(file, width = width, height = height, useDingbats = FALSE)
  on.exit(grDevices::dev.off(), add = TRUE)
  eval.parent(substitute(expr))
}

## ==============================================================
```

For Review Only

```
## 4. SEPA CORE STATISTICS FUNCTION
## ==============================================================
sepa_stats_all <- function(B_ref, F_ref,
                           planes = list(c(1, 2), c(3, 4)),
                           pid    = NULL) {
  stopifnot(is.matrix(B_ref), is.matrix(F_ref),
            ncol(B_ref) == ncol(F_ref))
  n_loc <- nrow(F_ref); p_loc <- nrow(B_ref)
  if (is.null(pid)) pid <- seq_len(n_loc)

  ## Overall person-domain cosines across all K dims (n x p)
  C_all <- person_domain_cosines(F_ref, B_ref)

  ## Per-plane statistics
  plane_out <- lapply(seq_along(planes), function(r) {
    idx <- planes[[r]]
    B_r <- B_ref[, idx, drop = FALSE]
    F_r <- F_ref[, idx, drop = FALSE]
    S_r <- F_r %*% t(B_r)                  # segment profiles (n x p)
    w_r <- signed_w(B_r)
    list(idx   = idx,
         rho   = rowwise_cor(S_r, w_r),
         C_r   = person_domain_cosines(F_r, B_r))
  })

  ## rho long table: one row per person x plane
  rho_tbl <- do.call(rbind, lapply(plane_out, function(x) {
    data.frame(id    = pid,
               plane = paste0("(", paste(x$idx, collapse = ","), ")"),
               rho   = x$rho,
               stringsAsFactors = FALSE)
  }))

  ## C_all long: person x domain across all dims
  C_all_df   <- cbind(id = pid, as.data.frame(C_all, check.names = FALSE))
  C_all_long <- reshape(C_all_df,
                        varying   = 2:(p_loc + 1),
                        v.names   = "C_all",
                        timevar   = "domain",
                        times     = safe_names(C_all, "D"),
                        direction = "long")[, c("id","domain","C_all")]
  rownames(C_all_long) <- NULL

  ## C_plane long: person x domain x plane
  C_plane_long <- do.call(rbind, lapply(plane_out, function(x) {
    tmp  <- cbind(id = pid, as.data.frame(x$C_r, check.names = FALSE))
    long <- reshape(tmp,
                    varying   = 2:(p_loc + 1),
                    v.names   = "C_plane",
                    timevar   = "domain",
                    times     = safe_names(x$C_r, "D"),
                    direction = "long")[, c("id","domain","C_plane")]
    long$plane <- paste0("(", paste(x$idx, collapse = ","), ")")
    rownames(long) <- NULL
    long
  }))

  list(rho     = rho_tbl,
       C_all   = C_all_long,
       C_plane = C_plane_long)
}
```

For Review Only

```
## Plane-fit rho helper (for exemplar output)
rho_from <- function(F2, B2) {
  w <- as.numeric(scale(sign(B2[, 1]) * sqrt(rowSums(B2^2))))
  S <- F2 %*% t(B2)
  apply(S, 1, function(si) cor(si, w))
}

## ============================================================
## 5. LOAD & PREPARE DATA
## ============================================================
set.seed(seed_main)
raw_df <- read.csv(data_path, header = TRUE, check.names = FALSE)
X      <- as.matrix(raw_df[, !(names(raw_df) %in% "ID"), drop = FALSE])

## Natural-scale PC1 – elevation alignment (before ipsatization)
X_raw       <- scale(X, center = TRUE, scale = FALSE)
sv_raw      <- svd(X_raw)
b1          <- sv_raw$v[, 1]
pc1_var_pct <- (sv_raw$d[1]^2 / sum(sv_raw$d^2)) * 100
one         <- rep(1, ncol(X_raw))
cos_pc1_elev <- abs(sum(b1) / (sqrt(sum(b1^2)) * sqrt(sum(one^2))))
cat(sprintf("PC1–elevation alignment: cos\u03b8 = %.3f ; PC1 var = %.2f%%\n",
            cos_pc1_elev, pc1_var_pct))

## Standardise domain names (FR -> FL if needed)
if ("FR" %in% colnames(X) && !("FL" %in% colnames(X)))
  colnames(X)[colnames(X) == "FR"] <- "FL"
target_cols <- c("LT","ST","CP","AP","VP","CK","FL")
common      <- intersect(colnames(X), target_cols)
if (length(common) < 7)
  stop("Expecting 7 WJ-IV domains. Found: ", paste(common, collapse = ", "))
X       <- X[, target_cols, drop = FALSE]
domains <- colnames(X)
n       <- nrow(X)
p       <- ncol(X)

## Ipsatize
Xstar <- ipsatize(X)

## Reference SVD (rank 4)
ref_fit <- svd_row_isometric(Xstar, K = 4)
B_ref   <- ref_fit$B   # p x 4
F_ref   <- ref_fit$F   # n x 4

## ID vector                                           FIX [8]: unconditional
pid <- if ("ID" %in% colnames(raw_df)) raw_df$ID else seq_len(n)

## ============================================================
## 6. SEGMENT PROFILE RECONSTRUCTIONS
## ============================================================
reconstruct_S <- function(B_scope, F_scope) F_scope %*% t(B_scope)

domain_names <- get_domain_names(B_ref, fallback = domains)

K <- ncol(B_ref)

if (K < 2) stop("Need at least 2 dims for Plane 1.")
B12 <- B_ref[, 1:2, drop = FALSE]; F12_ref <- F_ref[, 1:2, drop = FALSE]
S_plane1 <- reconstruct_S(B12, F12_ref)
```

For Review Only

```
B34 <- NULL; F34_ref <- NULL; S_plane2 <- NULL; S_planes12 <- NULL
if (K >= 4) {
  B34        <- B_ref[, 3:4, drop = FALSE]
  F34_ref    <- F_ref[, 3:4, drop = FALSE]
  S_plane2   <- reconstruct_S(B34,              F34_ref)
  S_planes12 <- reconstruct_S(B_ref[, 1:4, drop = FALSE],
                              F_ref[, 1:4, drop = FALSE])
}
S_allK <- reconstruct_S(B_ref, F_ref)

## Save reconstruction CSVs
write_matrix_wide(S_plane1, pid, "SEPA_pred_segment_profiles_plane1.csv",  domain_names)
if (!is.null(S_plane2))
  write_matrix_wide(S_plane2,   pid, "SEPA_pred_segment_profiles_plane2.csv",
domain_names)
if (!is.null(S_planes12))
  write_matrix_wide(S_planes12, pid, "SEPA_pred_segment_profiles_planes1plus2.csv",
domain_names)

## Write allK only if it differs from planes1+2
same_as_12 <- !is.null(S_planes12) &&
  isTRUE(all.equal(S_allK, S_planes12, tolerance = 1e-12))
if (!same_as_12)
  write_matrix_wide(S_allK, pid, "SEPA_pred_segment_profiles_allK.csv", domain_names)

## Optional xlsx output
if (write_predictions_xlsx) {
  out_xlsx <- list(Plane12 = as.data.frame(S_plane1))
  out_xlsx$Plane12 <- cbind(ID = pid, out_xlsx$Plane12)
  if (!is.null(S_plane2)) {
    out_xlsx$Plane34 <- cbind(ID = pid, as.data.frame(S_plane2))
  }
  writexl::write_xlsx(out_xlsx, path = predictions_out_path)
  cat("Wrote per-plane reconstructions to:", predictions_out_path, "\n")
}

## ============================================================
## 7. SEPA STATISTICS (rho, C_all, C_plane)
## ============================================================
planes_list <- list(c(1, 2), c(3, 4))
out <- sepa_stats_all(B_ref, F_ref, planes = planes_list, pid = pid)
stopifnot(is.list(out), all(c("rho","C_all","C_plane") %in% names(out)))

head(out$rho);     head(out$C_all);     head(out$C_plane)

write.csv(out$rho,     "SEPA_rho_per_person_plane.csv",   row.names = FALSE)
write.csv(out$C_all,   "SEPA_cosines_all_domains.csv",    row.names = FALSE)
write.csv(out$C_plane, "SEPA_cosines_by_plane.csv",       row.names = FALSE)

## Write wide cosine CSVs
write_long_to_wide(out$C_all, "id", "domain", "C_all",
                   "SEPA_cosines_all_domains_wide.csv", prefix = "")
for (pl in unique(out$C_plane$plane)) {
  subdf <- out$C_plane[out$C_plane$plane == pl, c("id","domain","C_plane")]
  fname <- paste0("SEPA_cosines_domains_plane",
                  gsub("[^0-9,]+", "", pl), ".csv")
  write_long_to_wide(subdf, "id", "domain", "C_plane", fname, prefix = "")
}
write_long_to_wide(out$rho, "id", "plane", "rho",
                   "SEPA_rho_by_plane.csv", prefix = "rho_")
```

For Review Only

```
## ============================================================
## 8. PARALLEL ANALYSIS
## ============================================================
PA <- parallel_analysis_ipsatized(Xstar, B = 2000, Kmax = 10,
                                  conf = 0.95, seed = seed_main + 1)
cat("PA significant dims:", paste(PA$sig_dims, collapse = ", "), "\n")

## ============================================================
## 9. BOOTSTRAP — DOMAIN COORDINATES (BCa)
## ============================================================
stat_domain <- function(data, idx, ref_B, K = 4) {
  Xb     <- ipsatize(data[idx, , drop = FALSE])
  fit    <- svd_row_isometric(Xb, K = K)
  B_boot <- fit$B
  ## FIX [6]: drop=FALSE on all multi-column slices
  R12  <- procrustes2(B_boot[, 1:2, drop = FALSE], ref_B[, 1:2, drop = FALSE])
  R34  <- procrustes2(B_boot[, 3:4, drop = FALSE], ref_B[, 3:4, drop = FALSE])
  B12  <- B_boot[, 1:2, drop = FALSE] %*% R12
  B34  <- B_boot[, 3:4, drop = FALSE] %*% R34
  B_al <- cbind(B12, B34)
  b_vec <- as.vector(t(B_al))  # domain-major: (j1 d1..d4, j2 d1..d4, ...)
  len2  <- rowSums(B_al^2)
  c(b_vec, len2)
}

run_boot <- function(fun, R) {
  if (!use_parallel) {
    boot(data = X, statistic = fun, R = R,
         parallel = "no", stype = "i", sim = "ordinary")
  } else if (parallel_type == "snow") {
    cl <- makeCluster(ncores)
    on.exit(stopCluster(cl), add = TRUE)
    boot(data = X, statistic = fun, R = R,
         parallel = "snow", ncpus = ncores, cl = cl,
         stype = "i", sim = "ordinary")
  } else {
    boot(data = X, statistic = fun, R = R,
         parallel = "multicore", ncpus = ncores,
         stype = "i", sim = "ordinary")
  }
}

cat("Bootstrapping domain coordinates (B =", B_dom, ")...\n")
boot_dom <- run_boot(
  function(d, idx) stat_domain(d, idx, ref_B = B_ref, K = 4),
  R = B_dom
)

## Index helpers
dom_coord_index <- function(j, k) (j - 1L) * 4L + k   # j=1..7, k=1..4
len2_index      <- function(j)    28L + j              # 29..35

idx_all  <- c(1:28, 29:35)
cis_dom  <- boot_cis_all(boot_dom, type = c("bca","perc"),
                         level = alpha_ci, idx_vec = idx_all)
ests     <- apply(boot_dom$t, 2, mean)

dom_coords_df <- data.frame(
  domain = rep(domains, each = 4),
  dim    = rep(1:4, times = p),
  index  = mapply(dom_coord_index, rep(1:p, each = 4), rep(1:4, times = p)),
```

For Review Only

```
    est    = ests[1:28]
  )
  ci_map <- cis_dom[cis_dom$index %in% dom_coords_df$index, ]
  dom_coords_df$lwr <- ci_map$lwr[match(dom_coords_df$index, ci_map$index)]
  dom_coords_df$upr <- ci_map$upr[match(dom_coords_df$index, ci_map$index)]

  len2_df <- data.frame(
    domain = domains,
    index  = sapply(1:p, len2_index),
    est    = ests[29:35]
  )
  ci_len2       <- cis_dom[cis_dom$index %in% len2_df$index, ]
  len2_df$lwr   <- ci_len2$lwr[match(len2_df$index, ci_len2$index)]
  len2_df$upr   <- ci_len2$upr[match(len2_df$index, ci_len2$index)]
  marker_thr    <- 4 / p
  len2_df$marker_flag <- len2_df$lwr > marker_thr

  cat("\n=== Domain coordinates (Dims 1-4) BCa ", alpha_ci * 100, "% ===\n", sep = "")
  print(dom_coords_df)
  cat("\n=== Domain ||b||^2 BCa ", alpha_ci * 100, "%; marker threshold K/p = ",
      round(marker_thr, 3), " (K=4) ===\n", sep = "")
  print(len2_df)

  ## ============================================================
  ## 10. BOOTSTRAP — PER-PERSON COSINES (Percentile)
  ## ============================================================
  Xstar_full <- Xstar

  stat_person_cos <- function(data, idx, ref_fit, Xstar_full, K = 4) {
    Xb     <- ipsatize(data[idx, , drop = FALSE])
    fit    <- svd_row_isometric(Xb, K = K)
    B_boot <- fit$B
    ## FIX [6]: drop=FALSE on all multi-column Procrustes slices
    R12  <- procrustes2(B_boot[, 1:2, drop = FALSE], ref_fit$B[, 1:2, drop = FALSE])
    R34  <- procrustes2(B_boot[, 3:4, drop = FALSE], ref_fit$B[, 3:4, drop = FALSE])
    B12  <- B_boot[, 1:2, drop = FALSE] %*% R12
    B34  <- B_boot[, 3:4, drop = FALSE] %*% R34
    F12  <- Xstar_full %*% B12
    F34  <- Xstar_full %*% B34
    C12  <- person_domain_cosines(F12, B12)
    C34  <- person_domain_cosines(F34, B34)
    B_all <- cbind(B12, B34)
    F_all <- Xstar_full %*% B_all
    Call  <- person_domain_cosines(F_all, B_all)
    c(as.vector(C12), as.vector(C34), as.vector(Call))
  }

  cat("\nBootstrapping per-person cosines (B =", B_cos, ")...\n")
  boot_cos <- run_boot(
    function(d, idx) stat_person_cos(d, idx, ref_fit = ref_fit,
                                     Xstar_full = Xstar_full, K = 4),
    R = B_cos
  )
  stopifnot(ncol(boot_cos$t) == 3L * n * p)

  ## CI extractor for one person across all domains in a plane
  extract_row_cis <- function(person_row_index, plane = c("12","34","all")) {
    plane <- match.arg(plane)
    idxs  <- sapply(1:p, function(j)
      cos_index(person_row_index, j, plane, n, p))
    percentile_ci_mat(boot_cos$t[, idxs, drop = FALSE], level = alpha_ci)
```

For Review Only

```
}

## Reference point estimates
B12_ref  <- B_ref[, 1:2, drop = FALSE]
C12_ref  <- person_domain_cosines(Xstar %*% B12_ref, B12_ref)
B34_ref  <- B_ref[, 3:4, drop = FALSE]
C34_ref  <- person_domain_cosines(Xstar %*% B34_ref, B34_ref)
Call_ref <- person_domain_cosines(F_ref, B_ref)

## ID -> row index mapping
id_vec     <- pid
id_to_index <- function(id) which(id_vec == id)[1L]
idx_sel    <- sapply(target_ids, id_to_index)
if (any(is.na(idx_sel)))
  stop("IDs not found: ", paste(target_ids[is.na(idx_sel)], collapse = ", "))

## ============================================================
## 11. EXEMPLAR TABLES
## ============================================================

## Profile differentiation norms
norm_plane1 <- sqrt(rowSums((Xstar %*% B12_ref)^2))
norm_plane2 <- sqrt(rowSums((Xstar %*% B34_ref)^2))

norms_tbl <- data.frame(
  ID          = target_ids,
  Norm_Plane1 = round(norm_plane1[idx_sel], 4),
  Norm_Plane2 = round(norm_plane2[idx_sel], 4)
)
write.csv(norms_tbl, "person_plane_norms.csv", row.names = FALSE)
cat("\nSaved person-plane norms.\n"); print(norms_tbl)

## Sanity: sums of squared norms = plane inertia
cat(sprintf("Plane 1  S||F_i^(1)||^2 = %.3f  (expected d1^2+d2^2 = %.3f)\n",
            sum(norm_plane1^2), ref_fit$d[1]^2 + ref_fit$d[2]^2))
cat(sprintf("Plane 2  S||F_i^(2)||^2 = %.3f  (expected d3^2+d4^2 = %.3f)\n",
            sum(norm_plane2^2), ref_fit$d[3]^2 + ref_fit$d[4]^2))

## Plane-fit rho for exemplars
rho_plane1 <- rho_from(Xstar %*% B12_ref, B12_ref)
rho_plane2 <- rho_from(Xstar %*% B34_ref, B34_ref)

rho_tbl <- data.frame(
  ID         = target_ids,
  Rho_Plane1 = round(rho_plane1[idx_sel], 3),
  Rho_Plane2 = round(rho_plane2[idx_sel], 3)
)
write.csv(rho_tbl, "person_plane_fit_rho.csv", row.names = FALSE)
cat("Saved plane-fit rho.\n"); print(rho_tbl)

## Per-person cosine tables with percentile CIs
build_plane_table <- function(idx_vec_rows, ids,
                              which_plane = c("12","34","all")) {
  plane <- match.arg(which_plane)
  est_mat <- switch(plane,
                    "12"  = C12_ref,
                    "34"  = C34_ref,
                    "all" = Call_ref)
  plane_label <- switch(plane,
                        "12"  = "Plane 1 (Dims 1-2)",
                        "34"  = "Plane 2 (Dims 3-4)",
```

For Review Only

```
                      "all" = "All retained dims (Dims 1-4)")
  tbls <- lapply(seq_along(idx_vec_rows), function(k) {
    i   <- idx_vec_rows[k]; id <- ids[k]
    cis <- extract_row_cis(i, plane)
    data.frame(ID     = id, Domain = domains, Plane = plane_label,
               Cosine = as.numeric(est_mat[i, ]),
               CI_Lwr = as.numeric(cis[, 1]),
               CI_Upr = as.numeric(cis[, 2]),
               stringsAsFactors = FALSE)
  })
  do.call(rbind, tbls)
}

plane_labels <- c("12", "34", "all")
plane_tbls   <- setNames(
  lapply(plane_labels, function(pl)
    build_plane_table(idx_sel, target_ids, pl)),
  plane_labels
)

write.csv(plane_tbls[["12"]],  "person_domain_cosines_plane12_percentile.csv",  row.names =
FALSE)
write.csv(plane_tbls[["34"]],  "person_domain_cosines_plane34_percentile.csv",  row.names =
FALSE)
write.csv(plane_tbls[["all"]], "person_domain_cosines_all_percentile.csv",      row.names =
FALSE)
cat("\nSaved cosine CSVs for Plane 1, Plane 2, and All dims.\n")

## ==============================================================
## 12. DOMAIN-DOMAIN COSINES (per plane)
## ==============================================================
upper_pairs <- which(upper.tri(diag(p)), arr.ind = TRUE)
pair_labels <- apply(upper_pairs, 1, function(a)
  paste0(domains[a[1]], "-", domains[a[2]]))

cos_pairs_from_B <- function(B2) {
  num <- B2 %*% t(B2)
  den <- sqrt(rowSums(B2^2)) %o% sqrt(rowSums(B2^2))
  (num / den)[upper.tri(diag(nrow(B2)))]
}

cos12_point <- cos_pairs_from_B(B12_ref)
cos34_point <- cos_pairs_from_B(B34_ref)

reconstruct_B_from_vec <- function(v28) {
  B_al <- t(matrix(v28, nrow = 4, byrow = TRUE))   # p x 4
  list(B12 = B_al[, 1:2, drop = FALSE],
       B34 = B_al[, 3:4, drop = FALSE])
}

cos12_boot <- t(apply(boot_dom$t[, 1:28, drop = FALSE], 1, function(v) {
  B <- reconstruct_B_from_vec(v); cos_pairs_from_B(B$B12)
}))
cos34_boot <- t(apply(boot_dom$t[, 1:28, drop = FALSE], 1, function(v) {
  B <- reconstruct_B_from_vec(v); cos_pairs_from_B(B$B34)
}))

ci12 <- percentile_ci_mat(cos12_boot, level = alpha_ci)
ci34 <- percentile_ci_mat(cos34_boot, level = alpha_ci)

domdom12_df <- data.frame(Pair    = pair_labels,
```

For Review Only

```
                           Cosine  = cos12_point,
                           CI_Lwr  = ci12[, 1],
                           CI_Upr  = ci12[, 2])
domdom34_df <- data.frame(Pair    = pair_labels,
                           Cosine  = cos34_point,
                           CI_Lwr  = ci34[, 1],
                           CI_Upr  = ci34[, 2])
write.csv(domdom12_df, "domain_domain_cosines_plane12_percentile.csv", row.names = FALSE)
write.csv(domdom34_df, "domain_domain_cosines_plane34_percentile.csv", row.names = FALSE)
cat("Saved domain-domain cosine CSVs.\n")

## ============================================================
## 13. BIPLOT (base R, row-isometric)
##     Produces one PDF per plane: Plane 1 (Dims 1-2) and Plane 2 (Dims 3-4).
## ============================================================

data_path_biplot <- "wj4_fake.csv"
ids_highlight    <- c(724, 944, 1080, 2117)

## Load & ipsatize once; reuse for both planes
df_bp  <- read.csv(data_path_biplot, header = TRUE, check.names = FALSE)
has_id <- "ID" %in% names(df_bp)
if (has_id) {
  id_vec_bp <- df_bp$ID
  X_bp      <- as.matrix(df_bp[, setdiff(names(df_bp), "ID")])
} else {
  id_vec_bp <- seq_len(nrow(df_bp))
  X_bp      <- as.matrix(df_bp)
}
domains_bp <- colnames(X_bp)
Xstar_bp   <- sweep(X_bp, 1, rowMeans(X_bp), "-")

sv_bp   <- svd(Xstar_bp)
U_bp    <- sv_bp$u; d_bp <- sv_bp$d; V_bp <- sv_bp$v
var_pct <- round(100 * (d_bp^2) / sum(d_bp^2), 1)

## Arrow/label/person styling (shared across both planes)
a.scale        <- 35;    t.scale <- 40
arrow_col      <- "#1F4E79"
dom_lwd        <- 2.0;   arrow_len <- 0.14;  dom_cex <- 1.10
base_back_frac <- 0.015
others_col     <- grDevices::adjustcolor("grey60", alpha.f = 0.30)
others_cex     <- 0.70
hi_col         <- "red3"; hi_bg <- "white"; hi_cex <- 1.15; hi_lwd <- 1.2
id_cex         <- 0.95

## Core biplot function — called for each plane
draw_sepa_biplot <- function(p1, p2) {
  Fx <- U_bp[, p1] * d_bp[p1];  Fy <- U_bp[, p2] * d_bp[p2]
  Vx <- V_bp[, p1];              Vy <- V_bp[, p2]

  end_x <- a.scale * Vx;  end_y <- a.scale * Vy
  lab_x <- t.scale * Vx;  lab_y <- t.scale * Vy
  lens  <- sqrt(Vx^2 + Vy^2)
  Udir  <- cbind(Vx, Vy) / pmax(lens, .Machine$double.eps)
  back_step <- base_back_frac * max(abs(c(end_x, end_y)))
  start_x   <- -back_step * Udir[, 1]
  start_y   <- -back_step * Udir[, 2]

  xlab      <- sprintf("Dimension %d (%.1f%%)", p1, var_pct[p1])
  ylab      <- sprintf("Dimension %d (%.1f%%)", p2, var_pct[p2])
```

For Review Only

```
  xr        <- range(c(Fx, end_x, lab_x, 0))
  yr        <- range(c(Fy, end_y, lab_y, 0))
  xlim      <- xr * 1.08;  ylim <- yr * 1.08
  plane_num <- if (p1 == 1L && p2 == 2L) 1L else
               if (p1 == 3L && p2 == 4L) 2L else NA

  out_file <- sprintf("SEPA_Biplot_Plane%d%d.pdf", p1, p2)
  save_pdf(out_file, {
    op <- par(mar = c(4.5, 5, 3.5, 2) + 0.1)
    on.exit(par(op), add = TRUE)
    plot(NA, NA, xlim = xlim, ylim = ylim,
         xlab = xlab, ylab = ylab,
         cex.lab = 1.20, cex.axis = 1.0,
         main = sprintf("Plane %d (Dims %d\u2013%d)", plane_num, p1, p2),
         cex.main = 1.25, xaxs = "i", yaxs = "i", asp = 1)
    abline(h = 0, col = "grey75", lty = 2)
    abline(v = 0, col = "grey75", lty = 2)

    idx_hi  <- if (length(ids_highlight))
                 match(ids_highlight, id_vec_bp) else integer(0)
    idx_hi  <- idx_hi[!is.na(idx_hi)]
    idx_oth <- setdiff(seq_len(nrow(Xstar_bp)), idx_hi)

    if (length(idx_oth))
      points(Fx[idx_oth], Fy[idx_oth],
             pch = 16, cex = others_cex, col = others_col)

    arrows(start_x, start_y, end_x, end_y,
           length = arrow_len, angle = 28,
           lwd = dom_lwd, col = arrow_col, code = 2)

    ## NOTE [10]: highlighted persons nudged outward by t.scale/a.scale so they
    ## do not overlap domain arrow tips. Plotted positions differ from true
    ## biplot coordinates by this constant scaling factor.
    if (length(idx_hi)) {
      scale_fac     <- t.scale / a.scale
      Fx_hi         <- Fx; Fy_hi <- Fy
      Fx_hi[idx_hi] <- Fx[idx_hi] * scale_fac
      Fy_hi[idx_hi] <- Fy[idx_hi] * scale_fac
      points(Fx_hi[idx_hi], Fy_hi[idx_hi],
             pch = 21, cex = hi_cex, lwd = hi_lwd, col = hi_col, bg = hi_bg)
      text(Fx_hi[idx_hi], Fy_hi[idx_hi],
           labels = as.character(id_vec_bp[idx_hi]),
           pos = 3, offset = 0.48, cex = id_cex, col = hi_col, font = 2)
    }
    text(lab_x, lab_y, labels = domains_bp, cex = dom_cex, col = arrow_col)
    box()
  })
  cat(sprintf("Saved %s\n", out_file))
}

## Produce both planes
draw_sepa_biplot(1, 2)   # Plane 1: Dims 1-2
draw_sepa_biplot(3, 4)   # Plane 2: Dims 3-4

## =============================================================
## END
## =============================================================
```

For Review Only

3. Three Supplementary Tables (S1–S3)

Supplementary Table S1

Comparison of SEPA with Tucker/MDPREF, PCA biplots, and calibrated biplots

Criteria	Tucker (1960) /MDPREF (Carroll, 1972)	PCA Biplot (Gabriel, 1971)	Calibrated Biplots (Gower & Hand, 1996 Greenacre, 2010; Graffelman & van Eeuwijk, 2005)	SEPA (this paper)
Primary aim	Preference mapping: represent individuals and attribute vectors so dot products reflect preferences; visual interpretation typically on first plane.	General joint display of rows (points) and columns (vectors) for low-dimensional exploration.	Improve interpretability via scaling/calibration of vector lengths, axes, and predictions; software aids (e.g., calibrate).	Person-centered profiling for assessment: define plane-wise segment profiles and two correlations; provide rules to combine planes for within-person inference.
Data preprocessing emphasis	Typically column-centering/standardization; not focused on per-person level removal.	Flexible; user chooses centering/scaling depending on goal.	As above; focus on scaling choices for interpretation.	Mandatory ipsatization (row-centering) to remove level effects; emphasizes pattern (within-person shape).
Core geometry/estimator	Vector model of unfolding; often implemented via PCA on subject profiles.	SVD/PCA factorization; row- or column-metric preserving mappings.	Same SVD/PCA foundation with alternative scalings and calibration overlays.	Row-centered SVD with row-metric preserving biplots; formalized person-level outputs.
Representation in the plane	Individuals as points; attributes/items as	Rows as points;	As PCA biplot, with	Same mapping, but **defines**

For Review Only

	vectors; dot product indicates preference.	columns as vectors; projection/angles used informally.	calibrated axes and prediction scales.	person→domain projections as segment values and uses them as analyzable profile elements.
Person-level summary outputs	Visual proximity/dot products; no standardized per-plane person summaries.	Primarily visual; no canonical per-person profile vector defined.	Visual prediction/axes calibration; still no standardized person-level summaries.	**Segment profile** vector per plane; two summaries: domain–person cosine (per domain) and domain–segment correlation (per plane).
Handling partial matches to multiple patterns	Possible by inspection but typically interpreted on the first plane.	Possible by inspection; not formalized.	Easier visually via calibration but not formalized.	Explicit: graded evidence across planes via cosines and per-plane correlations; no forced single-profile assignment.
Combining multiple planes (K>2)	Rarely specified.	Not standardized; usually first plane only.	Not standardized; method focuses on a plane.	Plane aggregation with singular-value weights; optional residual 1-D axis when K is odd.
Dimension selection	Heuristic/variance explained.	Heuristic/variance explained.	As above.	Horn's parallel analysis for selecting K; then present planes in variance order.
Model assumptions	Geometric unfolding; no probabilistic mixture model.	Linear algebraic; no distributional model required.	As PCA biplot.	Linear algebraic; adds ipsatization constraint and standardized outputs (segment

For Review Only

				vectors, correlations).
Primary use cases	Consumer/attitude preference mapping; sensory analysis.	General exploratory multivariate analysis.	Exploratory visualization with calibrated axes/predictions.	Clinical/psychological assessment where within-person pattern (not level) guides interpretation.
What SEPA adds beyond each prior method	Formal person-centered preprocessing; explicit per-plane person summaries (segment vectors, two correlations) and cross-plane aggregation.	Converts visual rules into analyzable outputs and provides weighting across planes; row-centering is required.	Builds on calibrated displays by defining standardized person-level statistics and a reproducible pipeline.	nan

Note. his table summarizes how SEPA differs from classical vector models, PCA biplots, and calibrated biplots across core analytic dimensions. Whereas earlier methods primarily support **visual inspection**—using projections, dot products, or calibrated axes for exploratory interpretation—SEPA introduces person-level estimates with explicit variance properties. These include *segment profiles* (plane-wise projected patterns), *domain–person cosines* (facet-specific associations), and the *plane-fit index* (within-plane coherence). SEPA additionally formalizes row-centered preprocessing**,** marker-domain identification**,** and variance-weighted aggregation across multiple planes, none of which are standardized in earlier geometric frameworks.

For Review Only

Supplementary Table S2

Comparison of SEPA with single-axis profile analysis (Kim, 2024).

Feature	Kim's 2024 *Psychological Methods* Paper	SEPA (Current Submission)
Data preprocessing	Ipsatization to isolate pattern effects	Same, but extended to plane-wise projection
Analytical unit	Single dimension (axis)	Two-dimensional planes (biplots)
Person-level output	Singular vector loadings only	$c_{ij}^{(r)}$, $\rho_i^{(r)}$, plane-level projections
Focus	Algebraic justification of SVD for pattern extraction	Operationalization of within-person interpretation

Feature	Kim's 2024 ***Psychological Methods*** Paper	SEPA (Current Submission)
Visualization	Not emphasized; dimensional loadings only	Plane-based biplots and segment profiles
Aggregation	None	Cross-plane combination via singular-value weighting
Intended use	Theoretical method for pattern decomposition	Applied interpretive pipeline for within-person profiling

Note. This table contrasts SEPA with prior unidimensional LE–PE profile analysis. The earlier single-axis approach established the theoretical value of ipsatized SVD for extracting pattern effects, but it remained limited to a single latent dimension, provided no plane-wise geometry, and did not define person-level summaries beyond singular-vector loadings. SEPA extends this foundation to a two-dimensional, plane-based system, yielding analyzable outputs—segment profiles, domain–person cosines, plane-fit correlations—and supporting cross-plane synthesis using singular-value weights. SEPA therefore transforms the algebraic basis of the earlier method into a complete, operational framework for within-person profiling.

For Review

Supplementary Table S3

Raw Ipsatized Scores for Four Illustrative Individuals Across Seven WJ-IV Cognitive Domains

Person	LT	ST	CP	AP	VP	CK	FR	Σ
	Long-Term Retrieval	*Short-Term Working Mem.*	*Cognitive Processing Speed*	*Auditory Processing*	*Visual Processing*	*Comprehension Knowledge*	*Fluid Reasoning*	*Row Sum*
**#724**	**−9.40**	+2.94	**+8.56**	−8.00	−2.72	+5.04	+3.57	*−0.01*
**#944**	**−14.62**	**+17.10**	−11.49	+2.42	−13.24	+15.37	+4.46	*+0.00*
**#1080**	+9.57	**+12.54**	**−14.42**	+4.52	−5.44	−6.13	−0.62	*+0.02*
**#2117**	+0.39	−4.40	+5.73	+3.07	**+9.56**	+4.37	**−18.75**	*−0.03*

Note. Ipsatized scores are row-centered deviations $\left(x^*_{ij} = x_{ij} - x_{i\bullet}\right)$, where $x_{i\bullet}$ is each person's mean across all seven domains. All four individuals share the same raw mean score of 100 but occupy distinct positions in the SEPA biplots due to their differing within-person profile shapes. By construction, ipsatized scores sum to zero across domains for each person (shown in the Σ column; minor deviations from 0.00 reflect rounding). **Bold green** values indicate each person's peak domain (highest relative strength); **bold red** values indicate each person's valley domain (greatest relative weakness).
